# Plastid phylogenomics clarifies broad-level relationships in *Bulbophyllum* (Orchidaceae) and provides insights into range evolution of Australasian section *Adelopetalum*

**DOI:** 10.3389/fpls.2023.1219354

**Published:** 2024-05-24

**Authors:** Lalita Simpson, Mark A. Clements, Harvey K. Orel, Darren M. Crayn, Katharina Nargar

**Affiliations:** ^1^ Australian Tropical Herbarium, James Cook University, Cairns, QLD, Australia; ^2^ College of Science and Engineering, James Cook University, Cairns, QLD, Australia; ^3^ Centre for Australian National Biodiversity Research (joint venture between Parks Australia and Commonwealth Industrial and Scientific Research Organisation (CSIRO)), Canberra, ACT, Australia; ^4^ School of BioSciences, The University of Melbourne, Parkville, VIC, Australia; ^5^ National Research Collections Australia, Commonwealth Industrial and Scientific Research Organisation (CSIRO), Canberra, ACT, Australia

**Keywords:** ancestral area reconstruction, Australasia, Bulbophyllinae, *Bulbophyllum*, divergence time estimation, high-throughput sequencing, Orchidaceae, phylogenomics

## Abstract

The hyperdiverse orchid genus *Bulbophyllum* is the second largest genus of flowering plants and exhibits a pantropical distribution with a center of diversity in tropical Asia. The only *Bulbophyllum* section with a center of diversity in Australasia is sect. *Adelopetalum*. However, the phylogenetic placement, interspecific relationships, and spatio-temporal evolution of this section remain largely unclear. To infer broad-level relationships within *Bulbophyllum*, and interspecific relationships within sect. *Adelopetalum*, a genome skimming dataset was generated for 89 samples, which yielded 70 plastid coding regions and a nuclear ribosomal DNA cistron. For 18 additional samples, Sanger data from two plastid loci (*mat*K and *ycf*1) and nuclear ITS were added using a supermatrix approach. The study provided new insights into broad-level relationships in *Bulbophyllum*, including phylogenetic evidence for the non-monophyly of sections *Beccariana*, *Brachyantha*, *Brachypus*, *Cirrhopetaloides*, *Cirrhopetalum*, *Desmosanthes*, *Minutissima*, *Oxysepala*, *Polymeres*, and *Sestochilos*. Section *Adelopetalum* and sect. *Minutissima s.s.* formed a highly supported clade that was resolved as a sister group to the remainder of the genus. Divergence time estimations based on a relaxed molecular clock model placed the origin of *Bulbophyllum* in the Early Oligocene (ca. 33.2 Ma) and sect. *Adelopetalum* in the Late Oligocene (ca. 23.6 Ma). Ancestral range estimations based on a BAYAREALIKE model identified the Australian continent as the ancestral area of the sect. *Adelopetalum.* The section underwent crown diversification from the mid-Miocene to the late Pleistocene, predominantly in continental Australia. At least two independent long-distance dispersal events were inferred eastward from the Australian continent to New Zealand and to New Caledonia from the early Pliocene onwards, likely mediated by predominantly westerly winds of the Southern hemisphere. Retraction and fragmentation of the eastern Australian rainforests from the early Miocene onwards are likely drivers of lineage divergence within sect. *Adelopetalum* facilitating allopatric speciation.

## 1 Introduction

The hyperdiverse orchid genus *Bulbophyllum* Thouars (Epidendroideae) is the second largest genus of flowering plants, with more than 2,100 species, and exhibits exceptional morphological and ecological diversity (Frodin, 2004; Pridgeon et al., 2014; POWO, 2022). Species of this predominantly epiphytic genus occur in a wide range of tropical and subtropical habitats, from montane rainforests to dry deciduous forests, savanna woodlands, and rocky fields with shrubby vegetation (Pridgeon et al., 2014). *Bulbophyllum* is distributed pantropically, occupying all botanical continents defined by Brummitt et al. (2001), except Antarctica and Eurasia. The genus is most diverse on the botanical continent of tropical Asia (1562 species), also occurring on the botanical continents of Africa (305), temperate Asia (152), Southern America (88), the Pacific (49), Australasia (Australia and New Zealand; 35), and Northern America (7) (POWO, 2022). Centers of diversity are found in tropical Asia in the floristic regions of Malesia (667), Papuasia (656), and Afrotropics in the western Indian Ocean region, on the islands of Madagascar, and Mascarenes (218) (POWO, 2022).

The high number of species and complex patterns of morphological variation have presented significant challenges for resolving relationships in *Bulbophyllum*, which is reflected in the substantial taxonomic revisions proposed. Traditionally, the Bulbophyllinae Schltr. (tribe Dendrobieae Endl.) included the large genus *Bulbophyllum* along with smaller genera, such as *Cirrhopetalum* Lindl., *Drymoda* Lindl., *Pedilochilus* Schltr., *Sunipia* Buch.-Ham. ex Sm., and *Trias* Lindl. (Dressler, 1993; Garay et al., 1994; Szlachetko and Margonska, 2001). Recent revisions treat all genera within the subtribe Bulbophyllinae as a more broadly defined *Bulbophyllum* and recognize 97 sections within the genus (Pridgeon et al., 2014; Vermeulen et al., 2014). Molecular phylogenetic studies have largely focused on species from specific geographic regions, such as Madagascar and Mascarenes (Fischer et al., 2007; Gamisch et al., 2015), the Neotropics (Smidt et al., 2011; Smidt et al., 2013), and Peninsular Malaysia (Hosseini et al., 2012) or on taxonomic groups such as the *Cirrhopetalum* alliance (Hu et al., 2020), and few have taken a global perspective (e.g., Gamisch and Comes, 2019). These studies revealed a strong biogeographic pattern within the genus with four main clades that include species largely confined or endemic within one broader geographical area: 1) continental Africa, 2) Madagascar and the Mascarene Islands, 3) Southern America, or 4) Asia (Fischer et al., 2007; Smidt et al., 2011; Gamisch et al., 2015; Gamisch and Comes, 2019). The Southern American clade, the Madagascan clade, and the continental African clade together form a highly supported lineage (Fischer et al., 2007; Smidt et al., 2011; Smidt et al., 2013; Gamisch et al., 2015; Gamisch and Comes, 2019), in sister group position to the Asian clade (Fischer et al., 2007; Gamisch et al., 2015; Gamisch and Comes, 2019). Previous molecular phylogenetic studies have mainly elucidated relationships within Madagascan, continental African and Neotropical sections and within the *Cirrhopetalum* alliance (Fischer et al., 2007; Smidt et al., 2011; Gamisch et al., 2015; Hu et al., 2020). However, evolutionary relationships within the Asian clade, which also includes taxa from the Australasian and Pacific regions, are still poorly understood, and the monophyly of sections within this clade has remained largely untested within a phylogenetic framework.

The study of hyperdiverse groups such as *Bulbophyllum* requires a robust phylogenetic framework for assessing the monophyly of its infrageneric taxa. High-throughput sequencing approaches facilitate the establishment of such a framework phylogeny to clarify broader evolutionary relationships and assess the monophyly of infrageneric taxa and their trait evolution (Hassemer et al., 2019; Nargar et al., 2022; van Kleinwee et al., 2022). However, phylogenomic studies that provide insights into the broad-level evolutionary relationships within the Asian clade of *Bulbophyllum* are still lacking. This hampers progress in understanding the spatio-temporal diversification of evolutionary lineages and trait evolution within this highly diverse genus.

**Figure 1:**
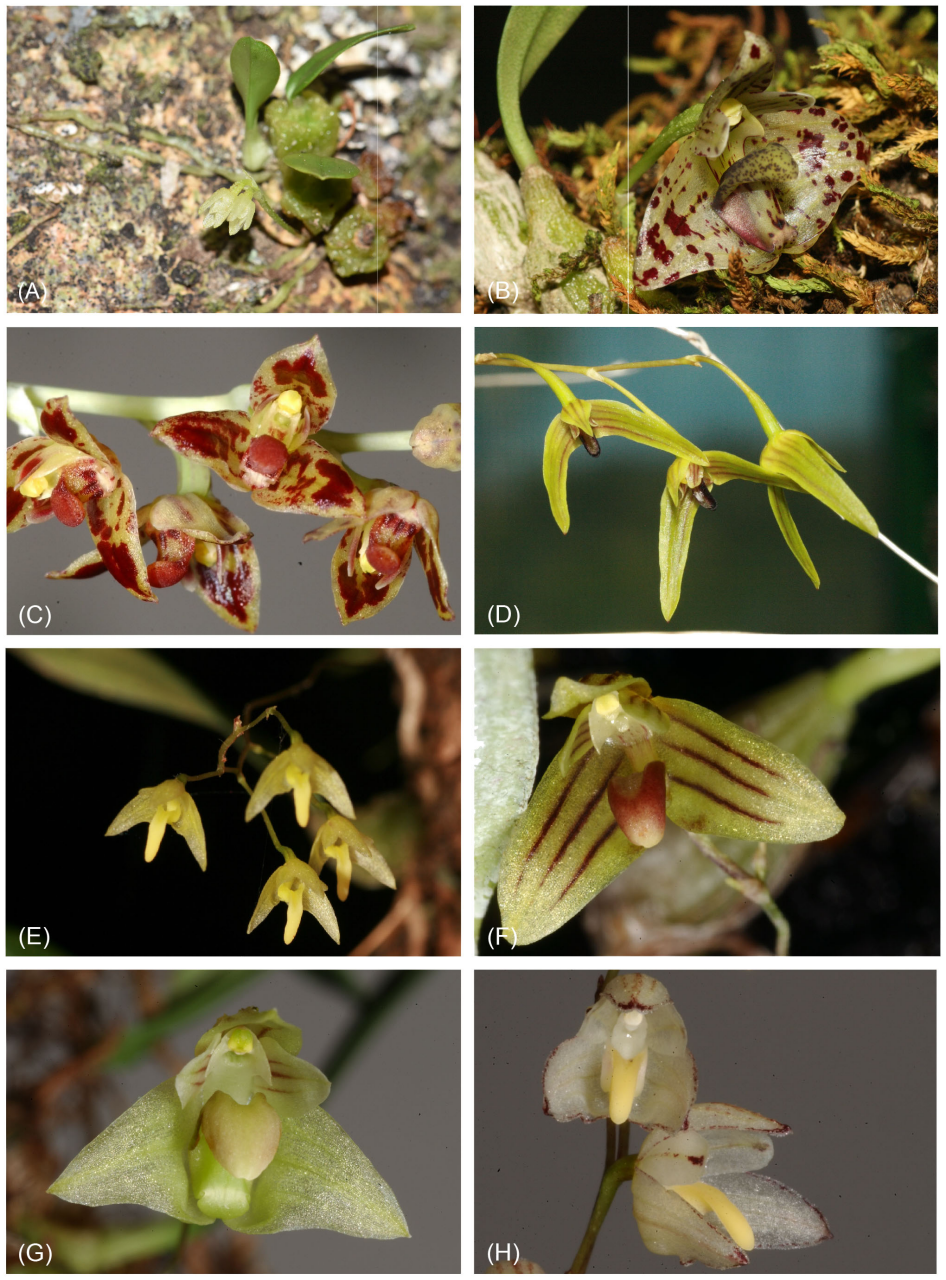
Morphological diversity within *Bulbophyllum* section *Adelopetalum*. **(A)** *B.* argyropus; **(B)** *B. weinthalii*; **(C)** *B. bracteatum*; **(D)** *B. elisae*; **(E)** *B. exiguum*; **(F)** *B. lilianae*; **(G)** *B. lageniforme*; **(H)** *B. newportii*. Photos M. Clements.

Within *Bulbophyllum*, section *Adelopetalum* has a unique distribution, being the only section with a center of diversity in Australia (Vermeulen, 1993; Brummitt et al., 2001; Pridgeon et al., 2014), and thus presents an interesting case study for range evolution within *Bulbophyllum*. This section comprises 12 tropical to temperate epi-lithophytic species (**Figure 1**). Nine species occur along Australia’s east coast in the montane forest communities of the Great Dividing Range, with one species (*B. argyropus*) found on Australian islands (Lord Howe Island and Norfolk Island). Two species (*B. corythium* and *B. lingulatum*) are endemic to the montane forests of New Caledonia, and one species (*B. tuberculatum*) is endemic to the lowland coastal forests of New Zealand. The section was circumscribed based on morphological affinities recognized among 10 species from Australia and New Caledonia previously assigned to the *Bulbophyllum* sections *Desmosanthes*, *Racemosae*, and *Sestochilus* (Dockrill, 1992; Vermeulen, 1993). Subsequent treatments identified two additional species within the *Adelopetalum* group, *B. weinthalii* and *B. exiguum* (Jones and Clements, 2002; Jones, 2020). Section *Adelopetalum* is characterized by thin creeping rhizomes adpressed to the host, anchored by filamentous roots with small pseudobulbs that are crowded to widely spaced, and a small single flat leaf arising from the apex of the pseudobulb. The inflorescence is single- to few-flowered, with small white, cream, or yellow flowers, sometimes with red or purple patterns. The petals are smaller than the sepals but similar in shape, with the bases of the lateral sepals fused to the column foot. The fleshly three-lobed labellum is firmly hinged to the apex of the column foot (Jones, 2020).

Previous cladistic analyses of the sect. *Adelopetalum*, based on morphological characters, resolved two main clades within the section, differentiated by the pseudobulb rib and ovary texture and the size and shape of the lower margin of the stelidia: the filiform column appendages typical for most *Bulbophyllum* (Vermeulen, 1993). Previous molecular phylogenetic studies based on the nuclear ribosomal ITS region (ITS1 + 5.8S + ITS2) have included two to three species from section *Adelopetalum* (Gamisch et al., 2015; Gamisch and Comes, 2019), placing them in an early diverging position within the Asian clade. However, the placement of sect. *Adelopetalum* remains unclear because of the lack of statistical support. Furthermore, phylogenetic relationships within sect. *Adelopetalum* and the evolution of its ancestral range are poorly understood and have not yet been investigated using molecular phylogenetic approaches.

This study aims to improve our understanding of the poorly understood evolutionary relationships among Asian and Australasian *Bulbophyllum* species and the spatio-temporal evolution of section *Adelopetalum*. The specific aims of this study were to 1) build a phylogenomic framework for *Bulbophyllum* with a focus on the Asian and Australasian species; 2) assess the monophyly and phylogenetic placement of sect. *Adelopetalum* within *Bulbophyllum*; 3) to infer interspecific relationships within sect. *Adelopetalum;* and 4) to reconstruct the range evolution of sect. *Adelopetalum*. The study sheds light on molecular systematics and evolution of Orchidaceae in the Australasian region over the Cenozoic.

## 2 Materials and methods

### 2.1 Sampling

A total of 136 orchid samples, representing 114 species, were included in the study. A broad sampling was included from the Asian, Australasian, and Pacific regions, representing 41 sections, i.e., 60% of sections recognized from these regions in the most recent treatment of the group (Pridgeon et al., 2014). From the Australasian region, all *Bulbophyllum* species were sampled (Australia: 30, New Zealand: 2). For *Bulbophyllum* sect. *Adelopetalum*, 28 samples were included, representing all 12 species recognized in the section. Morphologically closely related sect. *Minutissima* was included with nine samples representing five species comprising all four Australasian and Pacific species and two (of ca. 19) tropical Asian species. Sampling of five representative species from the Afrotropical clade was informed by previous phylogenetic studies (Fischer et al., 2007; Smidt et al., 2011; Gamisch and Comes, 2019). Species names followed the accepted taxonomy of Plants of the World Online (POWO, 2022). Sectional taxonomy followed the Internet Orchid Species Photo Encyclopedia (IOSPE, 2022), except for *B. exiguum*, which was placed in section *Adelopetalum*, and *B. wolfei*, which was placed in section *Polymeres* based on Jones and Clements (2002) and Jones (2020). The outgroup comprised representatives of subtribe Dendrobineae which is sister to Bulbophyllinae, and tribes Arethuseae, Malaxideae, Nervilieae, Neottieae based on previous molecular phylogenetic studies (Górniak et al., 2010; Givnish et al., 2015; Serna-Sánchez et al., 2021). Details of the plant material studied, voucher information, and the number of loci included for each sample are provided in **Supplementary Material S1**, and a complete list of loci analyzed is provided in **Supplementary Material S2**.

### 2.2 DNA extraction, amplification, and sequencing

Total genomic DNA was extracted from approximately 10 to 20 mg of silica-dried leaf material. Extractions were carried out with commercial extraction kits: Qiagen DNeasy plant kit (Venlo, Netherlands) and ChargeSwitch gDNA plant kit (Invitrogen, Carlsbad, USA), following the manufacturers’ protocols, or using the CTAB method (Doyle and Doyle, 1990), with modifications as described by Weising et al. (2005). Sequence data were generated using Sanger sequencing (46 samples) for the nuclear ribosomal ITS region (ITS1, 5.8s, ITS2) and two plastid genes (*mat*K, *ycf*1) and shotgun high-throughput sequencing (89 samples) to extract 70 plastid coding sequences (CDS) and the nuclear ribosomal DNA cistron (**Supplementary Material S2**) for subsequent analyses. Libraries for high-throughput sequencing were constructed from 50 ng to 100 ng of total DNA using the TruSeq Nano DNA LT library preparation kit (Illumina, San Diego, USA) for an insert size of 350 base pairs (bp) and paired-end reads according to the manufacturer’s protocol. Libraries were multiplexed with 96 indices and DNA sequencing with 125 bp paired-end reads was carried out on an Illumina HiSeq 2500 platform at the Australian Genomic Research Facility, Melbourne (Australia).

For Sanger sequencing, amplification of ITS was carried out with primers 17F and 26SER (Sun et al., 1994), *mat*K with primers 19F and 1326R (Gravendeel, et al., 2001; Cuénoud et al., 2002), and *ycf1* with primers 3720F, intR, intF, and 5500R (Neubig et al., 2009). The PCR protocol is described in **Supplementary Material S3**. Sequencing reactions were carried out using amplification primers, and sequencing was conducted on an AB3730xl 96-capillary sequencer (Australian Genome Research Facility, Brisbane, Australia).

### 2.3 Assembly and alignment

Sequences were assembled and edited in Geneious R10 (Kearse et al., 2012) using Geneious mapper. Illumina sequences were mapped to a reference set of plastid CDS extracted from *Dendrobium catenatum* (GenBank accession number KJ862886) and *ycf*68 from *Anoectochilus roxburghii* (GenBank accession number KP776980). Plastid coding regions were targeted in preference over whole plastomes to avoid challenges in aligning non-coding regions and to ensure homology across alignments that included representative sampling from the mega diverse genus *Bulbophyllum*. To build a closely related reference for the nuclear ribosomal ITS-ETS region, Illumina reads of *Bulbophyllum* sect. *Adelopetalum* representative *B. boonjee* (CNS_G07175) were first mapped to the ITS-ETS region of *Corallorhiza trifida* (JVF2676a). The *B. boonjee* Illumina reads were then mapped to the *B. boonjee* consensus sequence generated in the initial step to extend the region assembled to include the complete nuclear ribosomal DNA cistron (5´ETS, 18S, ITS1, 5.8S, ITS2, 28S, and 3´ETS). Illumina sequences for all the remaining samples were assembled against the *B. boonjee* nuclear ribosomal DNA cistron reference. Consensus sequences were generated with bases matching at least 60% of the total adjusted chromatogram quality, and a minimum coverage of ten reads. The quality of the assemblies was checked and edited manually where required. For Sanger sequencing, bidirectional reads were assembled using Geneious and manually edited. Additional sequences were sourced from DRYAD (https://doi.org/10.5061/dryad.n9r58) for *Coelogyne flaccida* (Givnish et al., 2015). DNA sequences were aligned using MAFFT v.7.222 (Katoh et al., 2002; Katoh et al., 2005) with default settings, visually inspected, and then concatenated into a nuclear and plastid supermatrix. The nuclear supermatrix included 136 accessions partitioned into coding and non-coding regions (alignment length: 6,341 bp, number of parsimony informative sites: 995 (16%)), and the plastid supermatrix included 130 accessions and 70 plastid coding regions, partitioned by gene and codon position (alignment length: 61,553 bp, number of parsimony informative sites: 5,789 (9%)). A plastid dataset comprising high-throughput sequencing data (excluding Sanger sequences) was analyzed separately, including 90 accessions and 70 plastid coding regions (alignment length: 61,553 bp, number of parsimony informative sites: 5,682 (9%)).

For divergence time estimations, the plastid supermatrix was reduced to one representative per species (indicated by an asterisk in **Supplementary Material S1**), comprising 111 accessions (alignment length: 60,984 bp, number of parsimony-informative sites: 5,755 (9%)). Edited ITS, matK and ycf1 generated from Sanger sequencing and raw Illumina reads used for this study have been deposited in the European Nucleotide Archive (ENA) at EMBL-EBI under accession number PRJEB72010 (https://www.ebi.ac.uk/ena/browser/view/PRJEB72010).

### 2.4 Phylogenetic analysis

Phylogenetic relationships were inferred using maximum likelihood in IQ-TREE v. 1.6.12 (Nguyen et al., 2015). The best-fit partition scheme and nucleotide substitution model for each partition were determined using IQ-TREE’s ModelFinder (Kalyaanamoorthy et al., 2017) based on the Akaike information criterion (AIC) (Akaike, 1974). Nodal support was assessed based on 1,000 replicates of ultrafast bootstrap approximation, with clades receiving >95 ultrafast bootstrap support (UFBS) considered as well supported (Minh et al., 2013; Hoang et al., 2018).

### 2.5 Divergence time estimation

The divergence times were estimated based on the plastid dataset in beast v. 2.4.8 (Bouckaert et al., 2014), applying the best-fit partition scheme and substitution model, as determined by IQ-TREE’s ModelFinder. We tested two molecular clock models: 1) strict clock (Zuckerkandl and Pauling, 1965) and 2) relaxed lognormal clock (Drummond et al., 2006), and two models of speciation and extinction: 1) Yule and 2) birth-death (Yule, 1925; Gernhard, 2008). Three secondary calibration points were used by applying priors with a normal distribution, mean ages, and 95% higher posterior density (HDP) intervals based on the results of a family wide molecular clock analysis by Chomicki et al. (2015). The root age was set at 55.02 Ma (HDP: 42.0–68.0). The next secondary calibration point was applied to the last common ancestor of Dendrobineae, Malaxideae, and Arethuseae and was set to 47.77 Ma (HDP: 36.4–59.1). Monophyly was constrained for this node, which is consistent with the relationships reconstructed in previous phylogenetic analyses (Chomicki et al., 2015; Givnish et al., 2015). The last secondary calibration was set at the stem node of Dendrobieae and Malaxideae with 38.68 Ma (HDP: 30.8–46.6). An additional calibration based on the fossil *Dendrobium winikaphyllum* (Conran et al., 2009) was applied to the stem node of the Australasian *Dendrobium* clade (*D. macropus*, *D. cunninghamii*, and *D. muricatum*), using a uniform distribution with an infinite maximum age and the minimum age constrained to 20.4 Ma, based on the minimum age of the strata containing the fossil (Mildenhall et al., 2014). Ten independent beast analyses were performed for 30 million MCMC generations, with trees sampled every 3 × 10^4^ generations. To assess the convergence of independent runs and determine burn-in fractions, log files were assessed using Tracer v.1.7.1 (Rambaut and Drummond, 2007). Log and tree files from independent runs were combined in LogCombiner (from the beast package) with a cumulative burn-in fraction of 10%–31%, and the sampling frequency was set to generate at least 10,000 tree and log files (Drummond and Bouckaert, 2015). The combined log file was assessed in Tracer to ensure that the effective sample size for all parameters was above 200. Five additional independent beast runs were conducted for the final analysis using a relaxed lognormal clock with birth death speciation to achieve an effective sample size above 200 for the ucldmean parameter. A maximum clade credibility tree was generated using TreeAnnotator (beast package) with median node heights. To compare the clock and speciation models, the Akaiki information criterion using the MCMC app from the beast package v 2.6.2 was used to measure the AICM for the combined MCMC runs generated in the beast analysis for each model (**Supplementary Material S4**).

### 2.6 Ancestral range analysis

The species distributions was extracted from POWO (2022). Biogeographic areas were largely delineated based on the botanical continents defined by Brummitt et al. (2001). The subcontinental regions of Papuasia, Australia, and New Zealand allowed a more fine-scale resolution of the range evolution in section *Adelopetalum* (Brummitt et al., 2001). The following seven biogeographic areas were coded: A) Africa, B) temperate Asia, C) tropical Asia, D) Papuasia, E) Australia, F) New Zealand, and G) Pacific. Ancestral ranges were estimated using RASP v. 4.0 (Yu et al., 2015) with the BioGeoBEARS package (Matzke, 2013), based on the maximum clade credibility tree obtained from the beast analysis of the plastid supermatrix, pruned of the outgroups to Dendrobieae. Three models of range evolution were tested: the dispersal-extinction cladogenesis model (DEC) (Ree and Smith, 2008), a maximum likelihood version of Ronquist’s parsimony dispersal-vicariance (DIVA) (Ronquist, 1997; Ronquist, 2001), termed DIVALIKE (Matzke, 2013), and a simplified likelihood interpretation of the Bayesian “BayArea” program (Landis et al., 2013), known as BAYAREALIKE (Matzke, 2013). No constraints were applied to the dispersal direction, and the maximum number of ranges was set to five based on the maximum number of observed areas of extant species. Likelihood values were compared, and the model of best fit determined by the AIC score (Akaike, 1974) was used to infer the marginal probabilities of alternative ancestral ranges at each node in the phylogeny (**Supplementary Material S5**).

## 3 Results

### 3.1 Phylogenetic relationships

#### 3.1.1 Phylogenetic relationships—plastid data

**Figure 2:**
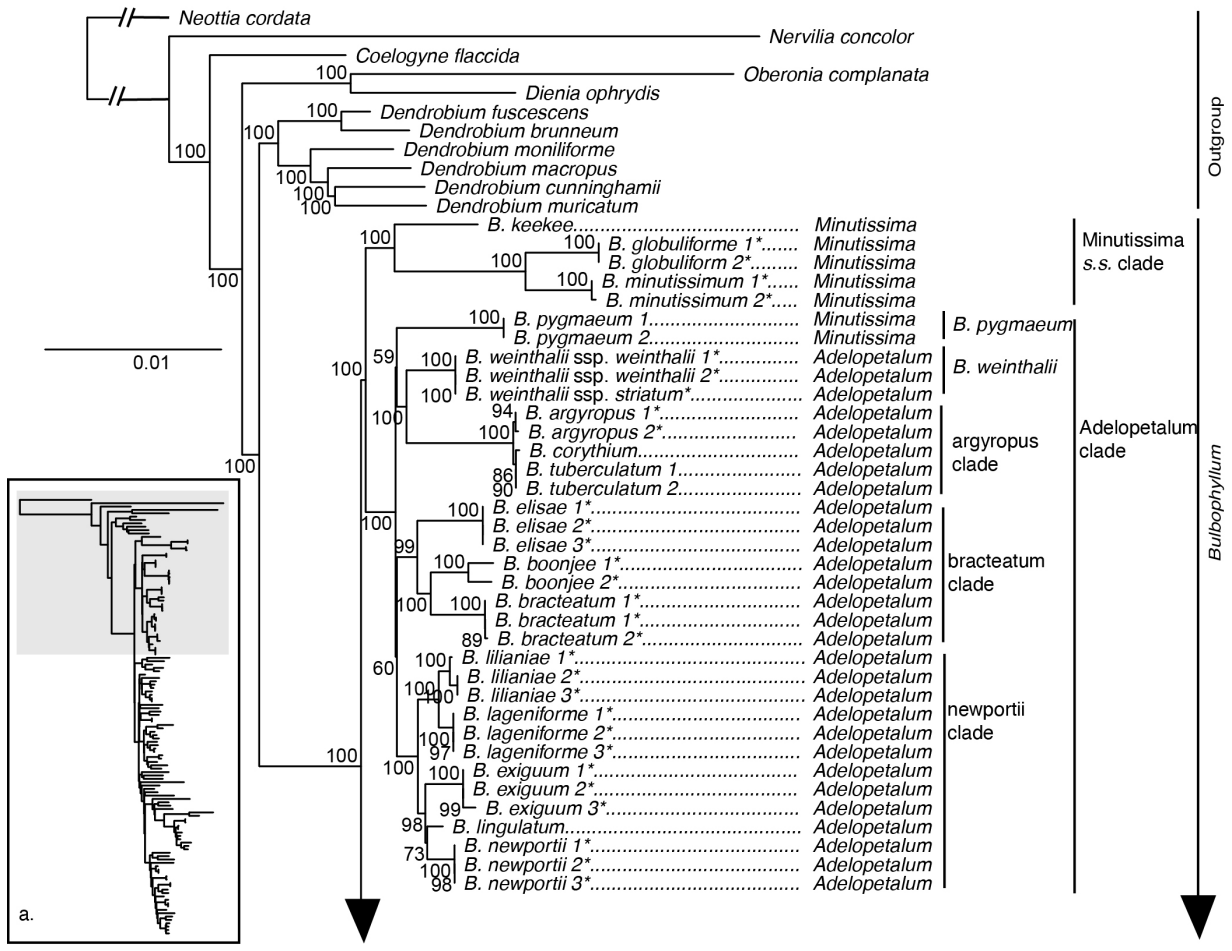
Maximum likelihood phylogenetic reconstruction of *Bulbophyllum* based on the supermatrix of 70 plastid coding regions in inset a. with *Bulbophyllum* sections *Adelopetalum* and *Minutissima* s.s. in detail. Ultrafast bootstrap values are given adjacent to nodes. Australian species are shown with an asterisk.

**Figure 3:**
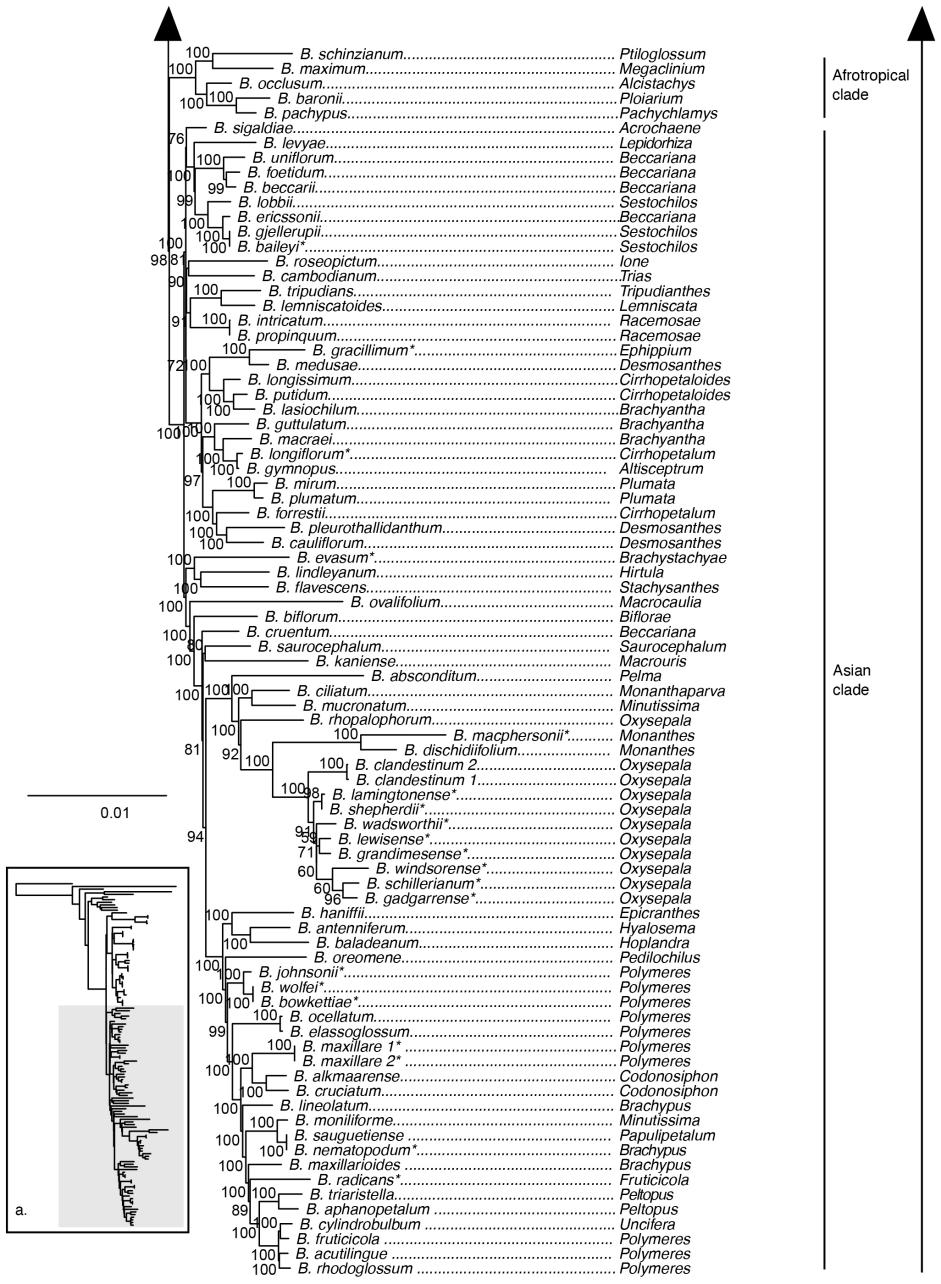
Maximum likelihood phylogenetic reconstruction of *Bulbophyllum* based on the supermatrix of 70 plastid coding regions in inset a. with Afrotropical and Asian *Bulbophyllum* clades in detail. Ultrafast bootstrap values are given adjacent to nodes. Australian species are shown with an asterisk.

The maximum likelihood phylogeny inferred from the 70 loci plastid supermatrix provided strong support for the monophyly of *Bulbophyllum* and its sister group relationship to *Dendrobium* (**Figure 2**). Sections *Adelopetalum* and *Minutissima s.s.* formed a highly supported clade, referred to as the Adelopetalum/Minutissima clade, which was resolved in the sister group position to the remainder of the genus (ultrafast bootstrap support/UFBS 98) (**Figures 2**, **3**). Within the Adelopetalum/Minutissima clade, all the *Adelopetalum* species and *B. pygmaeum* (sect. *Minutissima*) formed a highly supported lineage (UFBS 100) termed the Adelopetalum clade. Within the Adelopetalum clade several highly supported groups were resolved: 1) the argyropus clade consisting of *B. argyropus*, *B. corythium* and *B. tuberculatum* (UFBS 100), reconstructed in a highly supported sister group relationship to *B. weinthalii* (UFBS 100)*;* 2) the bracteatum clade, including *B. boonjee*, *B. bracteatum*, and *B. elisae* (UFBS 99); and 3) the newportii clade comprised of *B. exiguum*, *B. lageniforme*, *B. lilianae*, *B. lingulatum*, and *B. newportii* (UFBS 100). Relationships among the *B. pygmaeum*, argyropus clade + *B. weinthalii*, bracteatum, and newportii clades received weak support. The sister to the Adelopetalum clade was the highly supported Minutissimum clade comprising three species of sect. *Minutissima* (*B. globuliforme*, *B. keekee*, and *B. minutissimum*), including the type species of the section (UFBS 100) (**Figure 2**). Section *Minutissima* was identified as polyphyletic, with sect. *Minutissima* species placed within the Adelopetalum clade (*B. pygmaeum*) and Asian clade (*B. mucronatum* and *B. moniliforme*). Within the Asian clade, sections *Beccariana*, *Brachyantha*, *Brachypus*, *Cirrhopetaloides*, *Cirrhopetalum*, *Desmosanthes*, *Oxysepala*, *Polymeres*, and *Sestochilos* were identified as polyphyletic or paraphyletic. The phylogenetic relationships described here based on the plastid supermatrix (**Figures 1**, **3**) are supported by reconstructions based on the 70 gene plastid dataset (**Supplementary Material S6**).

Our analyses showed that sect. *Adelopetalum* does not share a close relationship with other Australasian *Bulbophyllum* species, such as those in sect. *Brachypus (B. nematopodum)*, sect. *Brachystachyae (B. evasum)*, sect. *Cirrhopetalum (B. longiflorum)*, sect. *Ephippium (B. gracillimum)*, sect. *Monanthes (B. macphersonii)*, sect. *Oxysepala (B. gadgarrense*, *B. grandimesense*, *B. lamingtonense*, *B. lewisense*, *B. schillerianum*, *B. shepherdii*, *B. wadsworthii*, *B. windsorense)*, sect. *Polymeres (B. bowkettiae*, *B. johnsonii*, *B. radicans*, and *B. wolfei)*, and sect. *Sestochilus (B. baileyi).* Australian species from each of these sections were placed at nine different positions within the Asian clade. Australian species from sect. *Polymeres* formed a highly supported clade (UFBS 100). Australian species from sect. *Oxysepala* formed a moderately supported clade (UFBS 91) and together with the type species of section *Oxysepala* from Papuasia (*B. cladistinum*) formed a close relationship with the Australian representative of section *Monanthes* (*B. macphersonii*) (UFBS 100).

#### 3.1.2 Phylogenetic relationships—nuclear data

**Figure 4:**
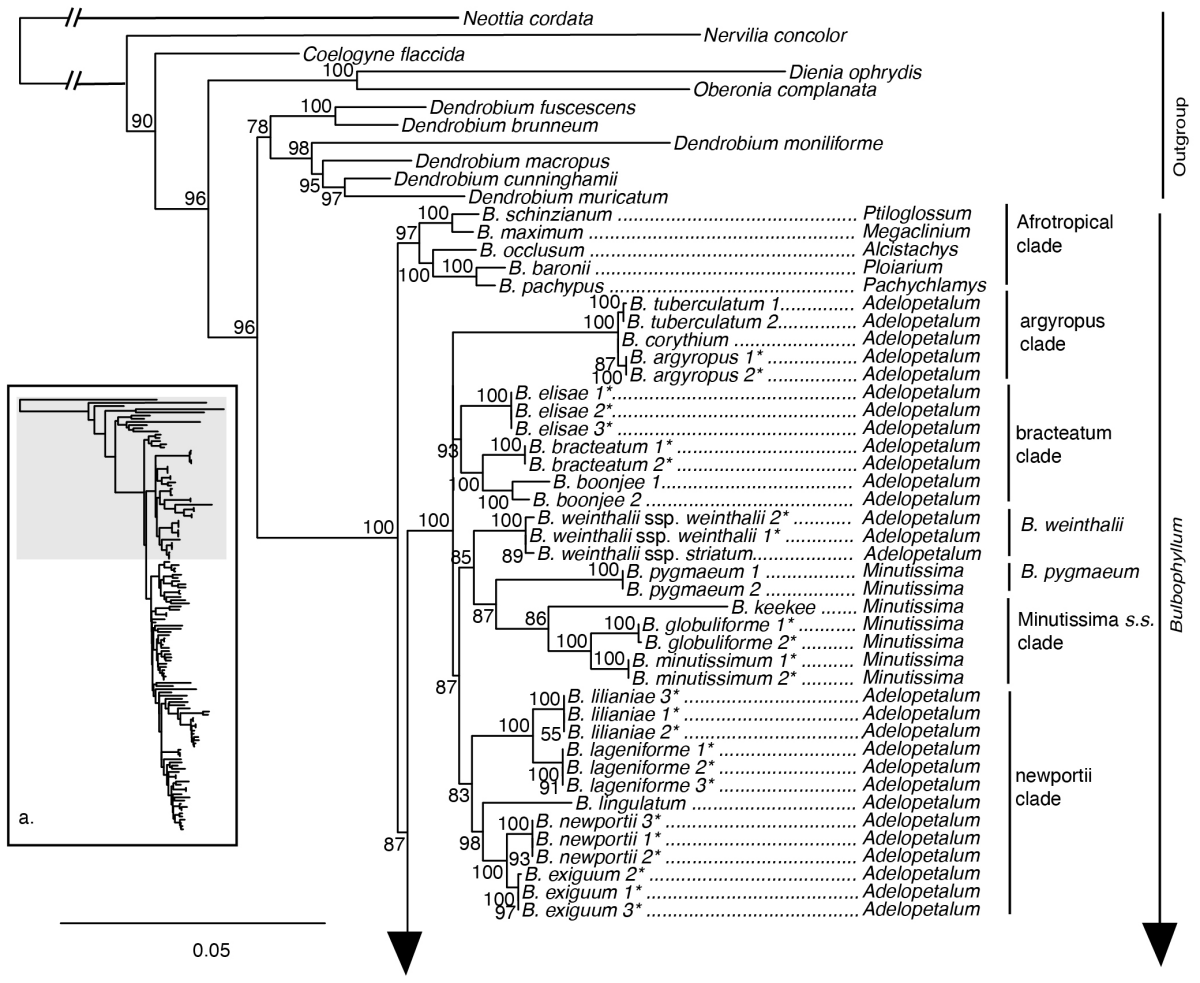
Maximum likelihood phylogenetic reconstruction of *Bulbophyllum* based on the nuclear ribosomal DNA cistron (5’ETS, 18s, ITS1, 5.8s, ITS2, 28s, 3’ETS) in inset a. with *Bulbophyllum* sections *Adelopetalum* and *Minutissima* in detail. Ultrafast bootstrap values are given adjacent to nodes. Australian species are shown with an asterisk.

**Figure 5:**
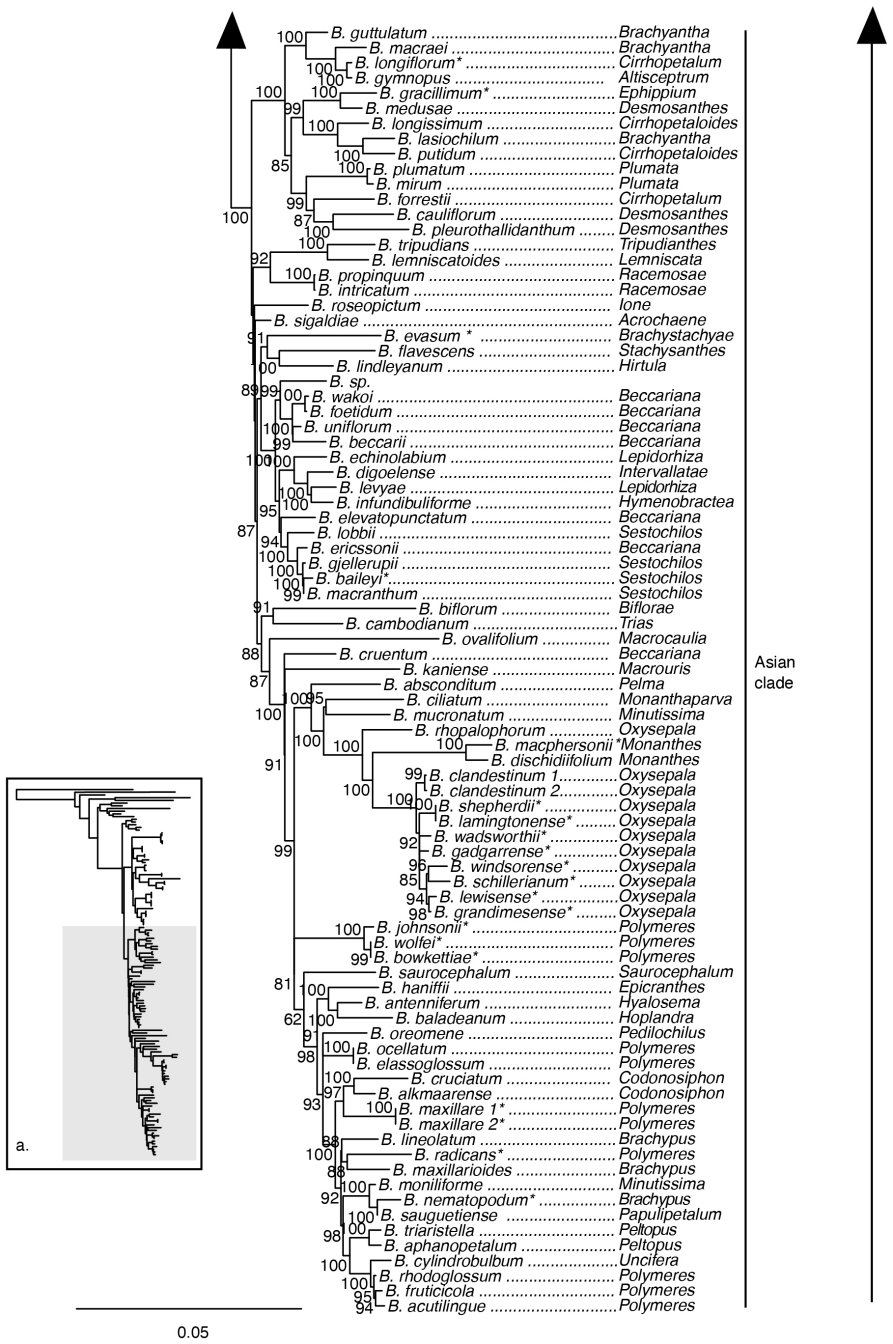
Maximum likelihood phylogenetic reconstruction of *Bulbophyllum* based on nuclear ribosomal DNA cistron (5’ETS, 18s, ITS1, 5.8s, ITS2, 28s, 3’ETS) in inset a. with Afrotropical and Asian *Bulbophyllum* clades in detail. Ultrafast bootstrap values are given adjacent to nodes. Australian species are shown with an asterisk.

The maximum likelihood phylogeny based on the nuclear ribosomal DNA cistron was resolved with overall lower support compared to analyses based on the 70 plastid loci supermatrix (**Figures 4**, **5**). Relationships among the outgroup taxa were concordant with the plastid phylogeny, and *Bulbophyllum* was resolved with maximum support. Within *Bulbophyllum*, the Afrotropical (UFBS 97), Asian (UFBS 100), and Adelopetalum/Minutissima (UFBS 100) clades were resolved with high to maximum support; however, their relationships were poorly supported. Within the Adelopetalum/Minutissima clade, the highly supported clades revealed in the plastid phylogeny were also reconstructed based on the nuclear dataset (argyropus clade (UFBS 100), bracteatum clade (UFBS 93), minutissimum clade (UFBS 86), and newportii clade (UFBS 83), however relationships among these remained poorly supported. Similar to reconstructions based on the plastid phylogeny: relationships among the argyropus, bracteatum and newportii clades, *B. pygmaeum* and *B. weinthalii* were poorly supported; sections *Beccariana, Brachyantha, Brachypus, Cirrhopetaloides, Cirrhopetalum, Desmosanthes, Minutissima, Oxysepala, Polymeres*, and *Sestochilos* were identified as polyphyletic or paraphyletic; and Australian species from sect. *Brachystachyae*, sect. *Cirrhopetalum*, sect. *Monanthes*, sect. *Oxysepala*, sect. *Brachypus*, sect. *Polymeres*, sect. *Ephippium*, sect. *Stenochilus* were placed in nine clades across the Asian clade.

### 3.2 Divergence time estimation

**Figure 6:**
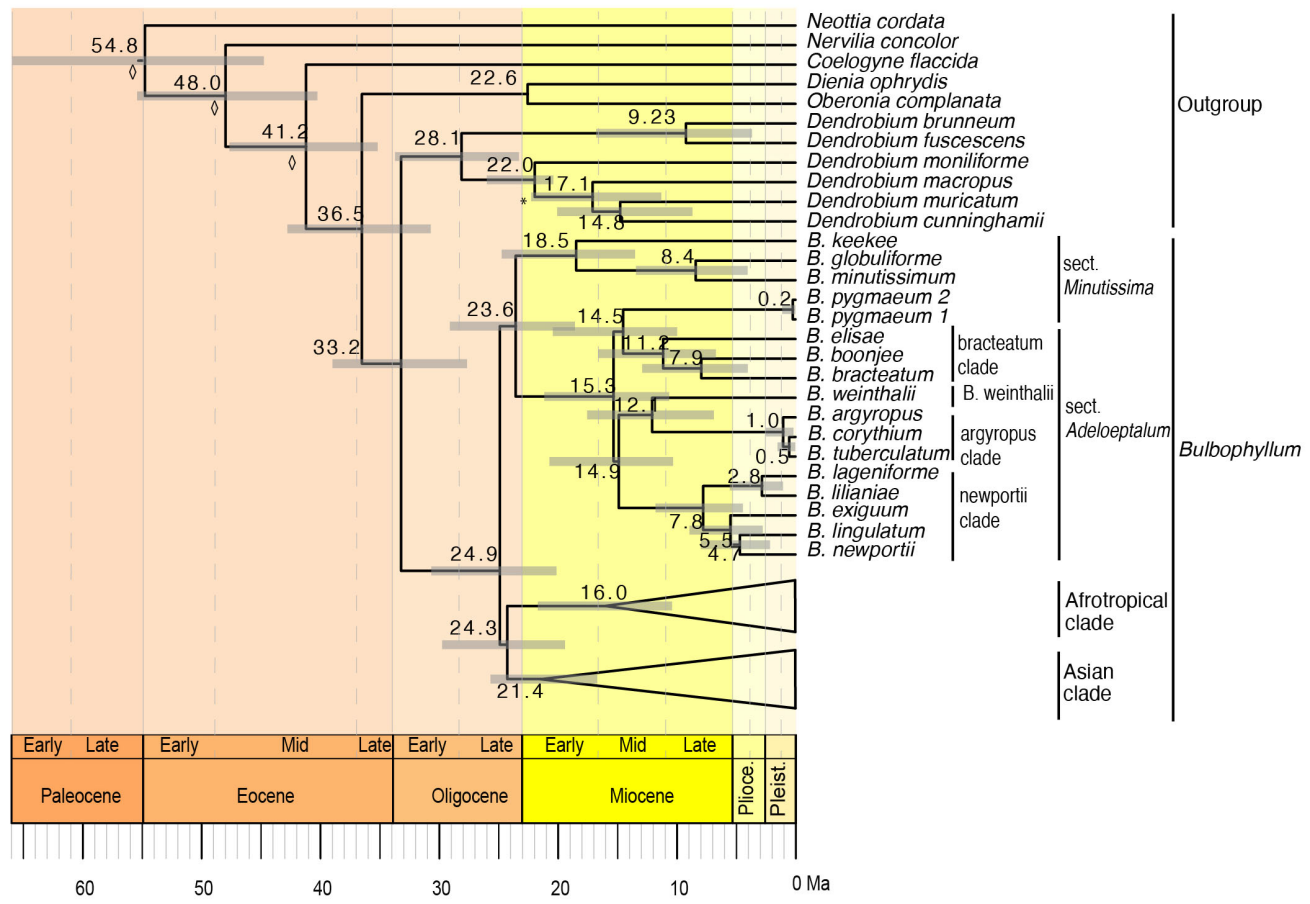
Maximum clade credibility chronogram for *Bulbophyllum* sect. *Adelopetalum* based on 70 plastid coding sequences, relaxed lognormal clock and birth death prior. Divergence dates and 95% highest posterior density values are indicated adjacent to nodes. Gray bars indicate 95% highest posterior density. The asterisk denotes the node constrained with a fossil calibration point; the diamond shape denotes nodes that were constrained by secondary calibration points.

The divergence time analysis based on a relaxed lognormal clock and birth death prior to speciation and extinction, which was identified as the model of best fit based on the Akaike information criterion (**Supplementary Material S4**), is presented here (**Figure 6**), with the Asian and Afrotropical clades collapsed and the complete chronogram provided in **Supplementary Material S7**. The divergence time analysis based on the plastid dataset was well-resolved and highly supported (**Figure 6**, **Supplementary Material S7**). The divergence between *Bulbophyllum* and *Dendrobium* was estimated to have occurred during the Early Oligocene, ca. 33.2 Ma (95% highest posterior probability density, HPD: 27.7–39.0). The crown of *Bulbophyllum*, constituting the divergence of the Adelopetalum/Minutissima clade from the remainder of the genus, was dated to the late Oligocene, ca. 24.9 Ma (HPD: 20.1–30.7). The divergence between the Asian clade and the Afrotropical clade was estimated to have occurred during the Late Oligocene, ca. 24.3 Ma (HPD: 19.4–29.8) and diversification within the Asian clade was estimated to have occurred from the mid Miocene 21.4 Ma (HPD: 17.2–26.4 Ma) and the Afrotropical clade from 16.0 Ma (HPD: 10.4–21.7). The crown age of the Adelopetalum/Minutissima clade was dated to the late Oligocene, ca. 23.6 Ma (HPD: 18.6–29.1), with a split of the Minutissimum clade from the Adelopetalum clade. The crown age of the Adelopetalum clade was dated to mid-Miocene, ca. 15.3 Ma (HPD: 10.6–21.2). The stem branches of major lineages within the Adelopetalum clade were estimated to have diversified during the mid-Miocene; the bracteatum clade was dated to ca. 14.5 Ma (HPD: 10.0–20.4); the lineage giving rise to *B. weinthalii* to ca. 12.1Ma (6.9–17.6); the argyropus clade to ca. 12.1 Ma (6.9–17.6); and the newportii clade to ca. 14.9 Ma (HPD 10.3–20.7). Diversification among species within these lineages occurred from the mid-Miocene onwards, with the most recent divergence identified during the late Pleistocene among *B. argyropus*, *B. corythium*, and *B. tuberculatum.*

### 3.3 Ancestral range analysis

**Figure 7:**
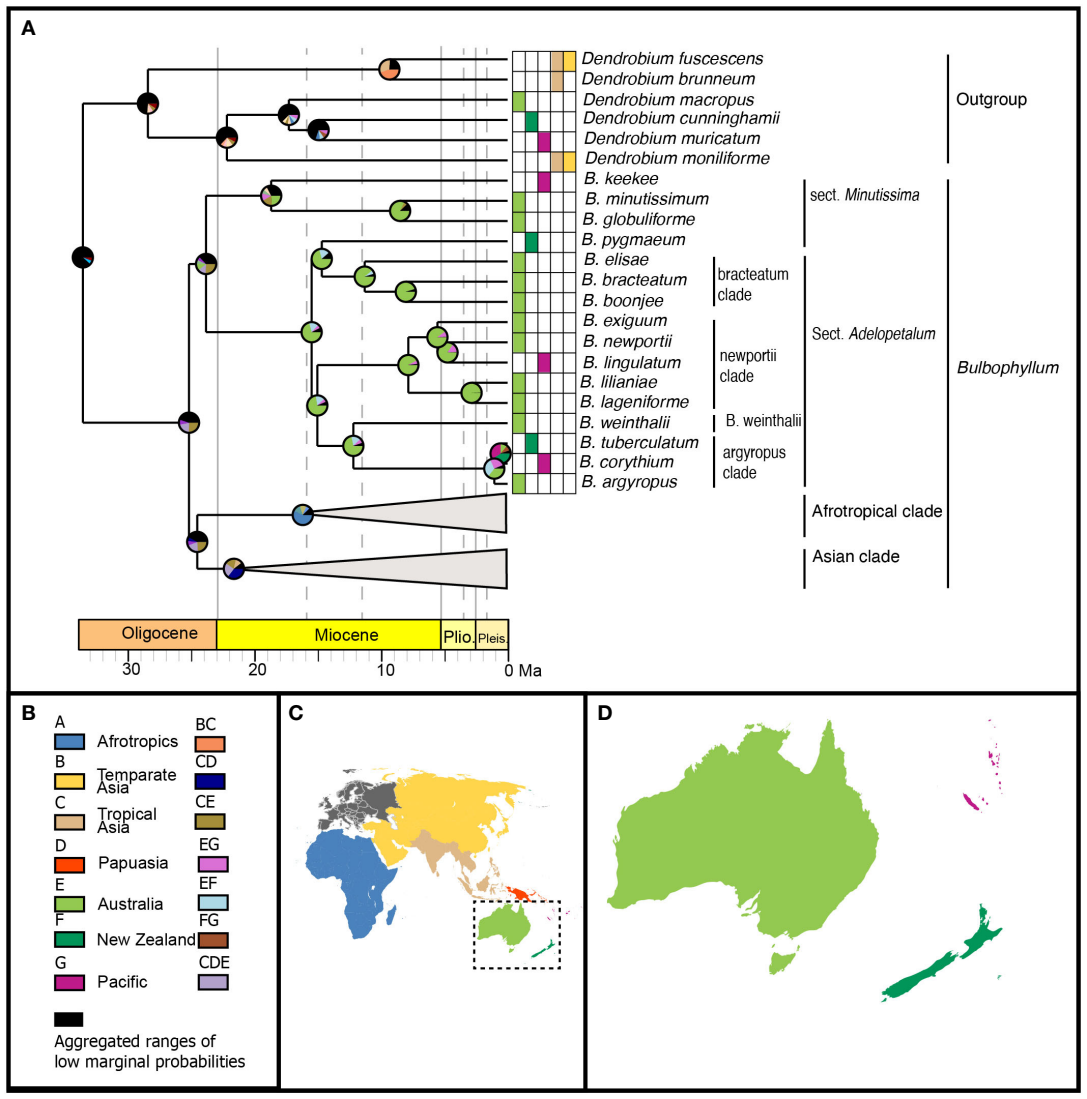
Range evolution of *Bulbophyllum* sect. *Adelopetalum*. **(A)** ancestral area reconstruction based on the BAYAREALIKE model with species extant distributions shown within the grid and pie charts at internal nodes representing marginal probabilities for alternative ancestral areas; **(B)** legend of color coded geographic regions and shared ancestral areas; **(C)** world map of color coded geographic regions delineated in the biogeographic analysis; **(D)** detail of Australasian color coded geographic regions.

Model testing of the three biogeographic models (DEC, DIVALIKE, BAYAREALIKE) using the Akaike information criterion identified the BAYAREALIKE model as the model of best fit for ancestral range estimation (**Supplementary Material S5**). The ancestral ranges estimated using the BAYAREALIKE model are presented here, with the Asian and Afrotropical clades collapsed (**Figure 7**). The complete chronogram is provided in **Supplementary Material S8**, the marginal probabilities for ancestral ranges at all nodes in **Supplementary Material S9**, and the node IDs in **Supplementary Material S10**.

Australia was reconstructed as the most likely ancestral range for the most recent common ancestor (MRCA) of the Adelopetalum clade (range probability (RP) 82) and all nodes within this lineage (RP 72–99), except for the argyropus clade (**Figure 7**). Range shifts from Australia were inferred from the early Pliocene to the Pacific region (New Caledonia) in the newportii clade, which gave rise to *B. lingulatum*. Range shifts were also inferred from Australia to the Pacific region (New Caledonia) and New Zealand, either in the lineage giving rise to MRCA of the argyropus clade or subsequently within this lineage. Three alternative ancestral ranges were reconstructed for the MRCA of the argyropus clade: Australia (RP 36) or widespread distributions, including Australia and New Zealand (RP 33) or Australia and New Caledonia (RP 26). Two alternative ranges were also reconstructed for MRCA of *B. corythium* and *B. tuberculatum*: New Zealand (RP 41) and New Caledonia (RP 34). Considering these alternative scenarios, range shifts within the argyropus clade were estimated to have occurred sometime between the mid-Miocene and the late Pliocene (12.1 Ma–0.5 Ma). The ancestral range of the MRCA of the Adelopetalum/Minutissima clade and *Bulbophyllum* remains unresolved in the ancestral range reconstruction. For the MRCA of the Adelopetalum/Minutissima clade, a wide ancestral range including Australia and tropical Asia received the highest relative probability (RP 26), while alternative ranges included a wide distribution across Australia, tropical Asia and Papuasia (RP 13) or Australia (RP 11).

## 4 Discussion

### 4.1 Phylogenetic relationships

This study provided a broad plastid phylogenetic framework for Asian and Australasian sections of *Bulbophyllum* and revealed a close relationship between sections *Adelopetalum* and *Minutissima s.s.*, that together form a highly supported early diverging lineage within the genus (**Figures 2**, **3**). Relationships based on 70 plastid genes support a sister group relationship between the *Adelopetalum*/*Minutissima* clade and the remainder of the genus (Asian + Afrotropical clades). Within the Adelopetalum/Minutissima clade, analyses based on our 70 plastid loci supermatrix showed a dichotomous split between the highly supported *Minutissima s.s.* and Adelopetalum clades. Species were reconstructed in each of these clades according to their sectional placement, except for the New Zealand endemic *B. pygmaeum* (sect. *Minutissima*), which was nested within the Adelopetalum clade, rendering the section *Adelopetalum* paraphyletic. Section *Minutissima* was identified as polyphyletic with the Australian (*B. minutissimum* (sect. type), *B. globuliforme*), and Pacific species (*B. keekee*) placed in the Minutissima clade and New Zealand species (*B. pygmaeum*) in the Adelopetalum clade, while the Asian species, *B. mucronatum* and *B. moniliforme* were resolved within the Asian clade. Section *Minutissima* has undergone numerous taxonomic changes with treatments ranging from a narrower circumscription recognizing species from Australia (Jones and Clements, 2001), to broader classifications, including 23 species from Thailand, Indonesia, Australia, New Zealand, New Caledonia, and New Guinea (Pridgeon et al., 2014). Our phylogenetic analysis based on plastid and nuclear markers did not reveal a close relationship between the sect. *Minutissima* species from the Australasian/Pacific region and Asian species *B. mucronatum* and *B. moniliforme*. Rather, in our analyses the Australasian species fell within the Adelopetalum/Minutissima clade while the Asian species were nested within the Asian clade. These results support the morphological studies that differentiate sect. *Minutissima* species from Australasia and Asia (Jones and Clements, 2001) and indicate minute pseudobulbs are a trait that has evolved more than once independently in the genus. Further studies, including dense sampling and ancestral character reconstruction, are required to assess the evolution of morphological traits and their phylogenetic utility across the Asian clade.

Within the section *Adelopetalum*, phylogenetic analyses supported current species concepts, except for species within the argyropus clade, which exhibited shallow genetic differentiation (**Figures 2**, **4**). Previous taxonomic treatments of this morphologically similar group have delineated up to three species: *B. argyropus* (Australia’s east coast and offshore islands: Lord Howe Island, and Norfolk Island), *B. corythium* (New Caledonia), and *B. tuberculatum* (New Zealand) (Halle, 1981; Vermeulen, 1993; Jones and Clements, 2002). Divergence dating analyses showed that this group represents a relatively recent radiation, reconstructing divergence among species during the Pleistocene. Further studies are required to clarify species delimitation and dispersal patterns utilising population-level sampling and genomic techniques suited to resolving relationships among recently diverged lineages, such as reduced representation high-throughput approaches like ddRAD, DArT or target sequence capture methods (Sansaloni et al., 2011; Peterson et al., 2012; Weitemier et al., 2014; Folk et al., 2015; Bagley et al., 2020; Schmidt-Lebuhn et al., 2022).

Previous cladistic analyses based on morphological traits of sect. *Adelopetalum* differentiated two main clades within the section, one comprising *B. lageniforme*, *B. lilianae*, *B. lingulatum*, and *B. newportii*, and the other uniting *B. argyropus*, *B. bracteatum*, and *B. elisae* (Vermeulen, 1993). Phylogenetic relationships based on plastid and nuclear markers found strong to moderate support for the first clade recovered in the cladistic analysis, corresponding to the newportii clade in the present analyses (**Figures 1**, **4**). The second group found in the cladistic analysis included three species placed in the phylogenetic analyses within either the bracteatum clade (*B. bracteatum*, *B. elisae*) or the argyropus clade (*B. argyropus*). However, the relationship between these two lineages remains unclear owing to low support. The sister group relationship between *B. bracteatum* and *B. argyropus* recovered in the cladistic analysis was not supported by phylogenetic reconstructions based on molecular data, suggesting that character states shared by these species, such as the number of veins in petals and number of pollinia, may be homoplasious. Further studies using ancestral character reconstruction are required to test the phylogenetic utility of the morphological traits used in prior studies.

While plastid phylogenomics has clarified the major clades and interspecific relationships within sect. *Adelopetalum* and broad-level relationships within *Bulbophyllum*, further studies are required. Non-monophyletic sections identified in the present study (e.g., sections *Beccariana*, *Brachyantha*, *Brachypus*, *Cirrhopetaloides*, *Cirrhopetalum*, *Desmosanthes*, *Minutissima*, and *Polymeres*) (**Figure 3**) and in previous molecular phylogenetic studies (Fischer et al., 2007; Smidt et al., 2011; Pridgeon et al., 2014; Hu et al., 2020) highlight the need for further taxonomic revision of *Bulbophyllum.* Further studies are required with an expanded sampling of diverse Asian and Pacific taxa to increase our understanding of evolutionary relationships and assess sectional classification in more detail. Phylogenetic relationships reconstructed from the nuclear ribosomal DNA cistron were not strongly supported, which is in line with previous molecular studies based on ITS (Gamisch et al., 2015; Gamisch and Comes, 2019; Hu et al., 2020). Approaches yielding a higher number of nuclear markers, such as target sequence capture, provide an opportunity to improve our understanding of evolutionary relationships in future studies. While assembling datasets with comprehensive species coverage within mega diverse groups, such as *Bulbophyllum*, remains a challenge, the present study provides an example of the use of a broad phylogenetic framework with targeted sampling within a section to test the monophyly and phylogenetic placement of groups of interest.

### 4.2 Spatio-temporal evolution of *Bulbophyllum* sect. *Adelopetalum*

Our divergence time analysis and ancestral range estimations showed that *Bulbophyllum* sect. *Adelopetalum* represents an Australasian lineage that originated on the Australian continent during the late Oligocene to early Miocene (**Figures 6**, **7**). The Australian ancestral range is largely conserved within the lineage, indicating that diversification among species has predominantly occurred on the Australian continent. The conservation of ancestral range observed within sect. *Adelopetalum* is consistent with previous phylogenetic analyses of *Bulbophyllum* that have shown a strong biogeographic signal among clades which are largely confined to biogeographic regions such as Madagascar, continental Africa and South America (Fischer et al., 2007; Smidt et al., 2011; Gamisch et al., 2015; Gamisch and Comes, 2019). The evolution of *Bulbophyllum* during the early Oligocene occurred subsequent to the breakup of Gondwana (Matthews et al., 2016; Zahirovic et al., 2016), indicating long-distance dispersal (LDD) in the evolution of biogeographical lineages within the genus (van den Berg, 2003; Smidt et al., 2011; Gamisch et al., 2015; Gamisch and Comes, 2019). Nevertheless, the conservation of ancestral ranges observed within the *Adelopetalum* lineage in this study and strong biogeographic signals among clades identified in previous studies indicate that LDD with successful establishment and persistence have been relatively infrequent within *Bulbophyllum*. Although the minute wind-dispersed seeds of orchids have a high dispersal potential, their successful establishment in a new area is limited by several factors, such as the presence of mycorrhizal partners necessary for germination and development, a suitable host or substrate and microclimatic conditions, and the availability of pollinators (Arditti and Ghani, 2000; Jersáková and Malinová, 2007; McCormick et al., 2012). Our results are consistent with those of previous studies that have identified *in situ* diversification as the dominant biogeographic process, despite evidence for LDD, and provides further support for the hypothesis that the complex requirements for successful establishment, rather than dispersal limitations, play an important role in constraining the geographic distribution of orchids (Givnish et al., 2016; Pérez-Escobar et al., 2017).

Our phylogenetic analyses further resolved the interspecific relationships in sect. *Adelopetalum* (**Figure 2**). Divergence time estimation showed that divergence among species occurred mainly during the Miocene and Pliocene (**Figure 6**), during a period of extensive changes in the distribution of forest vegetation on the Australian continent in response to drastic climatic changes. During the early Miocene, Australian vegetation diversified in response to the aridification of the Australian continent and the abrupt shift to a cool-dry climate during the mid-Miocene resulted in considerable fragmentation of rainforest habitats (Martin, 2006; Byrne et al., 2011). *Bulbophyllum* sect. *Adelopetalum* comprises epiphytic species that occur in mesic forest habitats; thus, diversification and fragmentation of these habitats were likely drivers of allopatric lineage divergence within this group. Sister group relationships were identified between two species pairs with disjunct distributions in Australia’s northern wet tropical rainforests and southeastern rainforests (*B. boonjee/B. bracteatum* and *B. newportii/B. exiguum*). These relationships support the hypothesis that the diversification and fragmentation of forest habitats in Australia have been important drivers of lineage divergence in Australia’s mesic biome (Byrne et al., 2011; Simpson et al., 2018).

While the ancestral range was predominantly conserved within the *Adelopetalum* lineage, range expansion events were inferred from continental Australia across the Coral and Tasman Seas to New Caledonia in the lineage giving rise to *B. lingulatum* and to New Zealand and New Caledonia in the argyropus clade (**Figure 7**). New Caledonia and New Zealand each have a long history of isolation from Australia that predates the evolution of *Bulbophyllum*, indicating that the colonization of these islands by *Bulbophyllum* species has been *via* LDD (Matthews et al., 2016). It remains unclear whether LDD to New Zealand and New Caledonia in the argyropus clade occurred from the early Miocene in the lineage, giving rise to the MRCA of the group or subsequently within this clade during the late Pleistocene; thus, the spatiotemporal evolution of this lineage requires further study.

The pattern of eastward dispersal observed in range shifts from Australia, across the Coral and Tasman Seas, is consistent with dispersal patterns inferred in other angiosperms, including *Abrotanella*, *Dendrobium*, *Dracophyllum*, *Korthalsella*, *Leucopogon*, *Northofagus*, *Oreobolus*, *Pterostylis*, *Rytidosperma*, and *Veronica* (Linder, 1999; Molvray et al., 1999; Lavarack et al., 2000; Swenson et al., 2001; Wagstaff et al., 2002; Chacón et al., 2006; Wagstaff et al., 2006; Albach and Meudt, 2010; Wagstaff et al., 2010; Puente-Lelièvre et al., 2013; Nargar et al., 2022). The bias towards eastward dispersal observed within section *Adelopetalum* among other plant groups may be facilitated by the predominant westerly winds occurring in the southern hemisphere that were initiated after the rifting of Australia and South America from Antarctica during the Eocene (Sanmartín et al., 2007).

### 4.3 Conclusions

This study provides an important phylogenomic framework for the mega genus *Bulbophyllum*, facilitating studies on trait and range evolution within the genus. Several Asian sections were resolved as paraphyletic, warranting taxonomic revision. Our plastid phylogenomic analyses revealed an early diverging lineage within *Bulbophyllum* composed of sect. *Adelopetalum* and sect. Minutissima *s.s*. For *Bulbophyllum* sect. *Adelopetalum*, this study reconstructed its origin in the early Oligocene and identified the Australian continent as the ancestral range. Species diversification within the section occurred predominantly on the Australian continent, with the fragmentation of mesic habitats during the Miocene identified as likely drivers of allopatric lineage divergence. Multiple independent long-distance dispersal events were inferred from the Australian continent eastward to the islands of New Zealand and New Caledonia.

## Data availability statement

The data used for this study have been deposited in the European Nucleotide Archive (ENA) at EMBL-EBI under accession number PRJEB72010 (https://www.ebi.ac.uk/ena/browser/view/PRJEB72010).

## Author contributions

Conceptualization: LS, KN, and MC; Data curation: LS and MC; Formal Analysis: LS and HO; Funding acquisition: LS, KN, DC, and MC; Investigation and Methodology: LS; Supervision: KN, DM, and MC; Writing—original draft: LS; Writing—review and editing: KN, HO, DM, and MC. All authors contributed to the article and approved the submitted version.
