# Supplementary material for: Plastid phylogenomics clarifies broad-level relationships in Bulbophyllum (Orchidaceae) and provides insights into range evolution of Australasian section Adelopetalum
Source: Front Plant Sci. 2024 May 24;14:1219354. doi: 10.3389/fpls.2023.1219354 (PMC11157511; doi:10.3389/fpls.2023.1219354)
Supplement: Supplementary file 1 [file DataSheet_1.docx]

Supplementary Material

Plastid phylogenomics clarifies broad-level relationships in *Bulbophyllum* (Orchidaceae) and provides insights into range evolution of Australasian section *Adelopetalum*

Lalita Simpson*, Mark A. Clements, Harvey K. Orel, Darren M. Crayn, Katharina Nargar

*** Correspondence:** Lalita Simpson: lalita.simpson1@jcu.edu.au

# Supplementary Material S1: Taxonomic, voucher and genomic marker details for plant material used in this study. Accessions included in divergence age estimations are indicated with an asterisk.

| Taxon | Section | Voucher details | DNA isolate | Markers |
| --- | --- | --- | --- | --- |
| *Bulbophyllum sigaldiae* Guillaumin (1955) | *Acrochaene* (Lindl.) J.J.Verm. Schuit. & de Vogel (2014) | Orchid Research Group 7679 (CANB) | CNS_G07581* | 70 plastid genes \| ITS_ETS cistron |
| *Bulbophyllum argyropus* 1 (Endl.) Rchb.f. (1876) | *Adelopetalum* (Fitzg.) J.J.Verm. (1993) | Orchid Research Group 7052 (CANB) | CNS_G03566* | 70 plastid genes \| ITS_ETS cistron |
| *Bulbophyllum argyropus* 2 (Endl.) Rchb.f. (1876) | *Adelopetalum* (Fitzg.) J.J.Verm. (1993) | D.Sinclair 5613 (CANB 892477.1) | CNS_G00333 | ITS \| matK \| ycf1 |
| *Bulbophyllum boonjee* 1 B.Gray & D.L.Jones (1984) | *Adelopetalum* (Fitzg.) J.J.Verm. (1993) | B.Gray 9761 (CNS 145935.1) | CNS_G07175* | 70 plastid genes \| ITS_ETS cistron |
| *Bulbophyllum boonjee* 2 B.Gray & D.L.Jones (1984) | *Adelopetalum* (Fitzg.) J.J.Verm. (1993) | D.L.Jones4226 (CBG 8913030) | CNS_G03563 | ITS \| matK \| ycf1 |
| *Bulbophyllum bracteatum* 1 (Fitzg.) F.M.Bailey (1891) | *Adelopetalum* (Fitzg.) J.J.Verm. (1993) | L.Simpson 497 (CNS 154146.1) | CNS_G01553* | 70 plastid genes \| ITS_ETS cistron |
| *Bulbophyllum bracteatum* 2 (Fitzg.) F.M.Bailey (1891) | *Adelopetalum* (Fitzg.) J.J.Verm. (1993) | Orchid Research Group 5159 (CANB) | CNS_G00383 | ITS \| matK \| ycf1 |
| *Bulbophyllum corythium* N.Hall (1981) | *Adelopetalum* (Fitzg.) J.J.Verm. (1993) | M.A.Clements 11238 (CANB 998252.1) | CNS_G00370* | ITS ycf1 |
| *Bulbophyllum elisae* 1 (F.Muell.) Benth. (1871) | *Adelopetalum* (Fitzg.) J.J.Verm. (1993) | L.Simpson 499 (CNS) | CNS_G01549 | ITS \| matK \| ycf1 |
| *Bulbophyllum elisae* 2 (F.Muell.) Benth. (1871) | *Adelopetalum* (Fitzg.) J.J.Verm. (1993) | L.Simpson 500 (CNS 154148.1) | CNS_G01550* | ITS \| matK \| ycf1 |
| *Bulbophyllum elisae* 3 (F.Muell.) Benth. (1871) | *Adelopetalum* (Fitzg.) J.J.Verm. (1993) | L.Simpson 498 (CNS 154147.1) | CNS_G01548 | ITS \| matK \| ycf1 |
| *Bulbophyllum exiguum* 1 F.Muell. (1860) | *Adelopetalum* (Fitzg.) J.J.Verm. (1993) | D.L.Jones19231 (CANB 667983.1) | CNS_G00322 | ITS \| matK \| ycf1 |
| *Bulbophyllum exiguum* 2 F.Muell. (1860) | *Adelopetalum* (Fitzg.) J.J.Verm. (1993) | L.Simpson 494 (CNS) | CNS_G01558* | ITS \| matK \| ycf1 |
| *Bulbophyllum exiguum* 3 F.Muell. (1860) | *Adelopetalum* (Fitzg.) J.J.Verm. (1993) | K.Schulte1 21 (CANB) | CNS_G00234 | ITS \| matK \| ycf1 |
| *Bulbophyllum lageniforme* 1 F.M.Bailey (1904) | *Adelopetalum* (Fitzg.) J.J.Verm. (1993) | L.Simpson LS167B (CNS) | CNS_G03707* | 70 plastid genes \| ITS_ETS cistron |
| *Bulbophyllum lageniforme* 2 F.M.Bailey (1904) | *Adelopetalum* (Fitzg.) J.J.Verm. (1993) | L.Simpson 180A (CNS) | CNS_G04503 | ITS \| matK \| ycf1 |
| *Bulbophyllum lageniforme* 3 F.M.Bailey (1904) | *Adelopetalum* (Fitzg.) J.J.Verm. (1993) | L.Simpson 95 (CNS 144295.1) | CNS_G01630 | ITS \| matK \| ycf1 |
| *Bulbophyllum lilianiae* 1 Rendle (1917) | *Adelopetalum* (Fitzg.) J.J.Verm. (1993) | L.Simpson 337E (CNS 154142.1) | CNS_G07754* | 70 plastid genes \| ITS_ETS cistron |
| *Bulbophyllum lilianiae* 2 Rendle (1917) | *Adelopetalum* (Fitzg.) J.J.Verm. (1993) | L.Simpson 496 (CNS) | CNS_G01561 | ITS \| matK \| ycf1 |
| *Bulbophyllum lilianiae* 3 Rendle (1917) | *Adelopetalum* (Fitzg.) J.J.Verm. (1993) | K.Schulte 87 (CNS) | CNS_G01632 | ITS \| matK \| ycf1 |
| *Bulbophyllum lingulatum* Rendle (1921) | *Adelopetalum* (Fitzg.) J.J.Verm. (1993) | M.A.Clements 7959 (CBG 9213395.1) | CNS_G06046* | 70 plastid genes \| ITS_ETS cistron |
| *Bulbophyllum newportii* 1 (F.M.Bailey) Rolfe (1909) | *Adelopetalum* (Fitzg.) J.J.Verm. (1993) | L.Simpson 155E (CNS 154141.1) | CNS_G03754* | 70 plastid genes \| ITS_ETS cistron |
| *Bulbophyllum newportii* 2 (F.M.Bailey) Rolfe (1909) | *Adelopetalum* (Fitzg.) J.J.Verm. (1993) | L.Simpson 495 (CNS 154145.1) | CNS_G01564 | ITS \| matK \| ycf1 |
| *Bulbophyllum newportii* 3 (F.M.Bailey) Rolfe (1909) | *Adelopetalum* (Fitzg.) J.J.Verm. (1993) | L.Simpson 492 (CNS 154143.1) | CNS_G01562 | ITS \| matK \| ycf1 |
| *Bulbophyllum tuberculatum* 1 Colenso (1884) | *Adelopetalum* (Fitzg.) J.J.Verm. (1993) | B.P.J. Molloy 112/99 (CANB) | CNS_G03993 | ITS \| matK \| ycf1 |
| *Bulbophyllum tuberculatum* 2 Colenso (1884) | *Adelopetalum* (Fitzg.) J.J.Verm. (1993) | 572204 (CHR 572204) | CHR572204* | ITS \| matK \| ycf1 |
| *Bulbophyllum weinthalii* ssp. *weinthalii* 1 R.S.Rogers (1933) | *Adelopetalum* (Fitzg.) J.J.Verm. (1993) | M.T.Mathieson 730 (BRI AQ0792800) | CNS_G06024* | 70 plastid genes \| ITS_ETS cistron |
| *Bulbophyllum weinthalii* ssp. *weinthalii* 2 R.S.Rogers (1933) | *Adelopetalum* (Fitzg.) J.J.Verm. (1993) | L.Simpson 493 (CNS 154144.1) | CNS_G01624 | ITS \| matK \| ycf1 |
| *Bulbophyllum weinthalii* ssp. *striatum* R.S. Rogers | *Adelopetalum* (Fitzg.) J.J.Verm. (1993) | Orchid Research Group 7186 (CANB) | CNS_G03564 | ITS \| matK \| ycf1 |
| *Bulbophyllum occlusum* Ridl., J. (1885) | *Alcistachys* Schltr. (1924) | Orchid Research Group 3376 (CANB 681129.1) | CNS_G07291* | 70 plastid genes \| ITS_ETS cistron |
| *Bulbophyllum gymnopus* Hook.f. (1890) | *Altisceptrum* J.J.Sm. (1914) | Orchid Research Group 7179 (CANB) | CNS_G05207* | 70 plastid genes \| ITS_ETS cistron |
| *Bulbophyllum beccarii* Rchb.f. (1879) | *Beccariana* Pfitz. (1889) | Orchid Research Group 7678 (CANB) | CNS_G07559* | 70 plastid genes \| ITS_ETS cistron |
| *Bulbophyllum cruentum* Garay, Hamer & Siegerist (1992) | *Beccariana* Pfitz. (1889) | Orchid Research Group 5524 (CANB) | CNS_G06039* | 70 plastid genes \| ITS_ETS cistron |
| *Bulbophyllum elevatopunctatum* J.J.Sm. (1920) | *Beccariana* Pfitz. (1889) | Orchid Research Group 7047 (CANB 948896.1) | CNS_G07187 | ITS_ETS cistron |
| *Bulbophyllum ericssonii* Kraenzl. (1893) | *Beccariana* Pfitz. (1889) | Orchid Research Group 6981 (CANB) | CNS_G05214* | 70 plastid genes \| ITS_ETS cistron |
| *Bulbophyllum foetidum* Schltr.(1913) | *Beccariana* Pfitz. (1889) | M.A.Clements 6431 (CANB) | CNS_G05873* | 70 plastid genes \| ITS_ETS cistron |
| *Bulbophyllum uniflorum* (Blume) Hassk. (1844) | *Beccariana* Pfitz. (1889) | Orchid Research Group 7670 (CANB) | CNS_G07571* | 70 plastid genes \| ITS_ETS cistron |
| *Bulbophyllum wakoi* Howcroft (1999) | *Beccariana* Pfitz. (1889) | Orchid Research Group 7176 (CANB) | CNS_G05222 | ITS_ETS cistron |
| *Bulbophyllum biflorum* Teijsm. & Binn. (1854) | *Biflorae* Garay, Hamer & Siegrist (1994) | Orchid Research Group 5887 (CANB) | CNS_G05442* | 70 plastid genes \| ITS_ETS cistron |
| *Bulbophyllum lasiochilum* C.S.P.Parish & Rchb.f. (1874) | *Brachyantha* Rchb.f 1861 | M.A.Clements 7343 (CANB 737416.1) | CNS_G07204* | 70 plastid genes \| ITS_ETS cistron |
| *Bulbophyllum guttulatum* (Hook.f.) N.P.Balakr. (1970) | *Brachyantha* Rchb.f. (1861) | Orchid Research Group 5587 (CANB) | CNS_G05250* | 70 plastid genes \| ITS_ETS cistron |
| *Bulbophyllum macraei* (Lindl.) Rchb.f. (1861) | *Brachyantha* Rchb.f. (1861) | M.A.Clements 12421 (CANB 956310.1) | CNS_G06045* | 70 plastid genes \| ITS_ETS cistron |
| *Bulbophyllum nematopodum* F.Muell. (1872) | *Brachypus* Schlechter (1913) | M.Harrison(CNS) | CNS_G01612* | ITS \| matK \| ycf1 |
| *Bulbophyllum lineolatum* Schltr. (1913) | *Brachypus* Schltr. (1913) | M.A.Clements 9580 (CANB) | CNS_G05262* | 70 plastid genes \| ITS_ETS cistron |
| *Bulbophyllum maxillarioides* Schltr. (1905) | *Brachypus* Schltr. (1913) | M.A.Clements 7253 (CBG 9013502.1) | CNS_G07332* | 70 plastid genes \| ITS_ETS cistron |
| *Bulbophyllum evasum* T.E.Hunt & Rupp (1950) | *Brachystachyae* Benth. & Hook.f. (1883). | B.Gray 8744 (CANB 599819.1) | CNS_G01247* | 70 plastid genes \| ITS_ETS cistron |
| *Bulbophyllum longissimum* (Ridl.) J.J.Sm. (1912) | *Cirrhopetaloides* Garay, Hamer & Siegerist (1994) | Orchid Research Group 5525 (CANB) | CNS_G06041* | 70 plastid genes \| ITS_ETS cistron |
| *Bulbophyllum putidum* (Teijsm. & Binn.) J.J.Sm. | *Cirrhopetaloides* Garay, Hamer & Siegerist (1994) | B.J.Wallace 19/91 (CANB) | CNS_G05872* | 70 plastid genes \| ITS_ETS cistron |
| *Bulbophyllum forrestii* Seidenf. (1974) | *Cirrhopetalum* (Lindl.) Rchb.f. (1861) | Orchid Research Group 1271 (CANB) | CNS_G06037* | 70 plastid genes \| ITS_ETS cistron |
| *Bulbophyllum longiflorum* Thouars (1822) | *Cirrhopetalum* (Lindl.) Rchb.f. (1861) | Orchid Research Group 7758 (CANB) | CNS_G06022* | 70 plastid genes \| ITS_ETS cistron |
| *Bulbophyllum alkmaarense* J.J.Sm. (1911) | *Codonosiphon* Schlechter 1913 | G.McCraith 076c (CANB) | CNS_G05454* | 70 plastid genes \| ITS_ETS cistron |
| *Bulbophyllum cruciatum* J.J.Sm. (1911) | *Codonosiphon* Schltr. (1911) | J.M.Taylor 2464 (CBG 8600516) | CNS_G01056* | 70 plastid genes \| ITS_ETS cistron |
| *Bulbophyllum cauliflorum* Hook.f. (1890) | *Desmosanthes* (Blume) J.J.Sm. (1933) | B.J.Wallace BJW 20/91 (CBG 9101909.1) | CNS_G05243* | 70 plastid genes \| ITS_ETS cistron |
| *Bulbophyllum medusae* (Lindl.) Rchb.f. (1861) | *Desmosanthes* (Blume) J.J.Sm. (1933) | Orchid Research Group 7684 (CANB) | CNS_G07573* | 70 plastid genes \| ITS_ETS cistron |
| *Bulbophyllum pleurothallidanthum* Garay (1999) | *Desmosanthes* (Blume) J.J.Sm. (1933) | Orchid Research Group 7681 (CANB) | CNS_G07567* | 70 plastid genes \| ITS_ETS cistron |
| *Bulbophyllum gracillimum* (Rolfe) Rolfe (1907) | *Ephippium* Schlechter 1913 | A.Field (CNS) | CNS_G01627* | ITS \| matK \| ycf1 |
| *Bulbophyllum haniffii* Carr (1932) | *Epicranthes* (Blume) Benth. & Hook.f. (1883) | Orchid Research Group 7677 (CANB) | CNS_G07566* | 70 plastid genes \| ITS_ETS cistron |
| *Bulbophyllum lindleyanum* Griff. (1851) | *Hirtula* Ridl. (1908), fide J.J.Verm. (2002) | Orchid Research Group 6237 (CANB) | CNS_G06035* | 70 plastid genes \| ITS_ETS cistron |
| *Bulbophyllum baladeanum* J.J.Sm. (1912) | *Hoplandra* J.J.Verm. (2008) | M.A.Clements 11187 (CANB 998208.1) | CNS_G05443* | 70 plastid genes \| ITS_ETS cistron |
| *Bulbophyllum antenniferum* (Lindl.) Rchb.f. (1861) | *Hyalosema* (Rolfe) Schltr. (1911) | T.Reeve 638 (CBG 7904522.1) | CNS_G05294* | 70 plastid genes \| ITS_ETS cistron |
| *Bulbophyllum infundibuliforme* J.J.Sm. (1906) | *Hymenobractea* Schltr. (1913) | M.A.Clements 6616 (CBG 9008257.1) | CNS_G07305 | ITS_ETS cistron |
| *Bulbophyllum digoelense* J.J.Sm. (1911) | *Intervallatae* Ridl. (1897) | Orchid Research Group 6246 (CANB) | CNS_G05276 | ITS_ETS cistron |
| *Bulbophyllum roseopictum* J.J.Verm., Schuit. & de Vogel | *Ione* [Lindley] J.J.Verm. Schuit. & de Vogel (2014) | Clements, M.A 12085 (CANB) | CNS_G03907* | 70 plastid genes \| ITS_ETS cistron |
| *Bulbophyllum lemniscatoides* Rolfe (1890) | *Lemniscata* Pfitz. (1888) | Orchid Research Group 7153 (CANB) | CNS_G05204* | 70 plastid genes \| ITS_ETS cistron |
| *Bulbophyllum levyae* Garay, Hamer & Siegerist (1995) | *Lepidorhiza* Schlechter (1911) | Orchid Research Group 7674 (CANB) | CNS_G07565* | 70 plastid genes \| ITS_ETS cistron |
| *Bulbophyllum echinolabium* J.J.Sm. (1934) | *Lepidorhiza* Schltr. (1911) | G.McCraith 165 (CANB) | CNS_G05237 | ITS_ETS cistron |
| *Bulbophyllum ovalifolium* (Blume) Lindl. (1830) | *Macrocaulia* (Blume) Aver. (1994) | Orchid Research Group 6254 (CANB) | CNS_G05210* | 70 plastid genes \| ITS_ETS cistron |
| *Bulbophyllum kaniense* Schltr. (1913) | *Macrouris* Schltr. (1913) | M.A.Clements 6249 (CANB) | CNS_G05254* | 70 plastid genes \| ITS_ETS cistron |
| *Bulbophyllum maximum* (Lindl.) Rchb.f. (1861) | *Megaclinium* (Lindl.) Summerh. (1921) | Orchid Research Group 6250 (CANB) | CNS_G05289* | 70 plastid genes \| ITS_ETS cistron |
| *Bulbophyllum globuliforme* 1 Nicholls (1938) | *Minutissima* Pfitz. (1888) | L.Simpson 501 (CNS 154149.1) | CNS_G01584 | ITS \| matK \| ycf1 |
| *Bulbophyllum globuliforme* 2 Nicholls (1938) | *Minutissima* Pfitz. (1888) | G.McCraith 86 (CANB) | CNS_G00392* | ITS \| matK \| ycf1 |
| *Bulbophyllum keekee* N.Hall (1977) | *Minutissima* Pfitz. (1888) | M.A.Clements 5675 (CBG 8916317.1) | CNS_G07329* | 70 plastid genes \| ITS_ETS cistron |
| *Bulbophyllum minutissimum* 1 (F.Muell.) F.Muell. (1878) | *Minutissima* Pfitz. (1888) | L.Simpson 503 (CNS 154151.1) | CNS_G01587* | 70 plastid genes \| ITS_ETS cistron |
| *Bulbophyllum minutissimum* 2 (F.Muell.) F.Muell. (1878) | *Minutissima* Pfitz. (1888) | L.Simpson 502 (CNS 154150.1) | CNS_G01585 | ITS \| matK \| ycf1 |
| *Bulbophyllum moniliforme* E.C.Parish & Rchb.f. | *Minutissima* Pfitz. (1888) | M.A.Clements 9605 (CANB) | CNS_G03763* | 70 plastid genes \| ITS_ETS cistron |
| *Bulbophyllum mucronatum* (Blume) Lindl. (1830) | *Minutissima* Pfitz. (1888) | M.A.Clements MAC 12379 (CANB) | CNS_G05440* | 70 plastid genes \| ITS_ETS cistron |
| *Bulbophyllum pygmaeum* 1 (Sm.) Lindl. | *Minutissima* Pfitz. (1888) | B.P.J. Molloy 062/98 (CANB) | CNS_G04599* | 70 plastid genes \| ITS_ETS cistron |
| *Bulbophyllum pygmaeum* 2 (Sm.) Lindl. | *Minutissima* Pfitz. (1888) | B.P.J. Molloy 134/99 (CANB) | CNS_G03988* | ITS \| matK \| ycf1 |
| *Bulbophyllum ciliatum* (Blume) Lindl. (1830) | *Monanthaparva* Ridl. 1896 | Orchid Research Group 4316 (CANB) | CNS_G05450* | 70 plastid genes \| ITS_ETS cistron |
| *Bulbophyllum dischidiifolium* J.J.Sm. (1909) | *Monanthes* (Blume) Aver. (1994) | M.A.Clements 6941 (CBG 9013189.2) | CNS_G05278 | 70 plastid genes \| ITS_ETS cistron |
| *Bulbophyllum macphersonii* Rupp (1934) | *Monanthes* (Blume) Aver. (1994) | K.Schulte85 (CANB) | CNS_G01039* | ITS \| ycf1 |
| *Bulbophyllum clandestinum* 1 Lindl. (1841) | *Oxysepala* Bentham & J D Hook.f. (1883) | T.M. Reeve 735 (CBG 8905730.1) | CNS_G07307* | 70 plastid genes \| ITS_ETS cistron |
| *Bulbophyllum clandestinum* 2 Lindl. (1841) | *Oxysepala* Bentham & J D Hook.f. (1883) | Orchid Research Group 3080 | CNS_G05272* | 70 plastid genes \| ITS_ETS cistron |
| *Bulbophyllum gadgarrense* Rupp (1949) | *Oxysepala* Bentham & J D Hook.f. (1883) | M.A.Clements 8535 (CANB) | CNS_G00933* | ITS \| ycf1 |
| *Bulbophyllum grandimesense* B.Gray & D.L.Jones (1989) | *Oxysepala* Bentham & J D Hook.f. (1883) | L.J. Roberts s.n. (CANB) | CNS_G01588* | ITS \| ycf1 |
| *Bulbophyllum lamingtonense* D.L.Jones (1993) | *Oxysepala* Bentham & J D Hook.f. (1883) | Orchid Research Group 6946 (CANB) | CNS_G01013* | ITS \| ycf1 |
| *Bulbophyllum lewisense* B.Gray & D.L.Jones (1989) | *Oxysepala* Bentham & J D Hook.f. (1883) | M.Harrison(CNS) | CNS_G01608* | ITS \| ycf1 |
| *Bulbophyllum rhopalophorum* Schltr. (1913) | *Oxysepala* Bentham & J D Hook.f. (1883) | M.A.Clements 6846 (CBG 9013094) | CNS_G05261* | 70 plastid genes \| ITS_ETS cistron |
| *Bulbophyllum schillerianum* Rchb.f. (1860) | *Oxysepala* Bentham & J D Hook.f. (1883) | P.Forster 24990 (CANB 755447.1) | CNS_G01597* | ITS \| ycf1 |
| *Bulbophyllum shepherdii* (F.Muell.) Rchb.f. (1871) | *Oxysepala* Bentham & J D Hook.f. (1883) | Orchid Research Group 4858 (CANB) | CNS_G00804* | ITS \| ycf1 |
| *Bulbophyllum wadsworthii* Dockrill (1964) | *Oxysepala* Bentham & J D Hook.f. (1883) | C.D. Kilgour 1143 (CNS 137212.1) | CNS_G00574* | ITS \| ycf1 |
| *Bulbophyllum windsorense* B.Gray & D.L.Jones (1989) | *Oxysepala* Bentham & J D Hook.f. (1883) | M.Harrison (CNS) | CNS_G01610* | ITS \| ycf1 |
| *Bulbophyllum pachypus* Schltr. (1924) | *Pachychlamys* Schltr. (1925) | M.A.Clements 12774 (CANB) | CNS_G07740* | 70 plastid genes \| ITS_ETS cistron |
| *Bulbophyllum sauguetiense* Schltr. (1913) | *Papulipetalum* Schltr. (1913) | M.A.Clements 7235 (CBG 9013484.1) | CNS_G05258* | 70 plastid genes \| ITS_ETS cistron |
| *Bulbophyllum oreomene* J.J.Verm., Schuit. & de Vogel (2014) | *Pedilochilus* (Schltr.) J.J.Ver. & P.O'Byrne (2011) | G. Hope s.n. (National University Australian) | CNS_G07517* | 70 plastid genes \| ITS_ETS cistron |
| *Bulbophyllum absconditum* J.J.Sm. (1905) | *Pelma* (Finet) Schltr. (1913) | M.A. Clements 11242 (CANB 998255.1) | CNS_G05247* | 70 plastid genes \| ITS_ETS cistron |
| *Bulbophyllum triaristella* Schltr. (1913) | *Peltopus* Schltr. (1913) | M.A.Clements s.n. (CANB) | CNS_G01202* | 70 plastid genes \| ITS_ETS cistron |
| *Bulbophyllum aphanopetalum* Schltr. (1906) | *Peltopus* Schltr. (1913) | P.Ziesing (CANB) | CNS_G05241* | 70 plastid genes \| ITS_ETS cistron |
| *Bulbophyllum baronii* Ridl. (1885) | *Ploiarium* Schlechter (1925) | Orchid Research Group 6242 (CANB) | CNS_G06040* | 70 plastid genes \| ITS_ETS cistron |
| *Bulbophyllum mirum* J.J.Sm. (1906) | *Plumata* J.J.Verm., Schuit. & de Vogel (2014) | Orchid Research Group 5501 (CANB 778009.1) | CNS_G06031* | 70 plastid genes \| ITS_ETS cistron |
| *Bulbophyllum plumatum* Ames (1915) | *Plumata* J.J.Verm., Schuit. & de Vogel (2014) | Orchid Research Group 7673 (CANB) | CNS_G07578* | 70 plastid genes \| ITS_ETS cistron |
| *Bulbophyllum acutilingue* J.J.Sm.(1908) | *Polymeres* (Blume) J.J.Verm. & O'Byrne (2008) | S.Rose. 25 (CANB) | CNS_G07612* | 70 plastid genes \| ITS_ETS cistron |
| *Bulbophyllum bowkettiae* F.M.Bailey (1884) | *Polymeres* (Blume) J.J.Verm. & O'Byrne (2008) | B.Gray 8697 (CANB 596002) | CNS_G00826* | ITS \| ycf1 |
| *Bulbophyllum elassoglossum* Siegerist, Amer. (1991) | *Polymeres* (Blume) J.J.Verm. & O'Byrne (2008) | M.A.Clements 11502 (CANB) | CNS_G05456* | 70 plastid genes \| ITS_ETS cistron |
| *Bulbophyllum fruticicola* Schltr. (1905) | *Polymeres* (Blume) J.J.Verm. & O'Byrne (2008) | T.M.Reeve 3372 (CBG 9000963.1) | CNS_G07481* | 70 plastid genes \| ITS_ETS cistron |
| *Bulbophyllum johnsonii* T.E.Hunt (1950) | *Polymeres* (Blume) J.J.Verm. & O'Byrne (2008) | C.D.Kilgour 150 (CNS) | CNS_G00538* | ITS \| ycf1 |
| *Bulbophyllum maxillare* 1 (Lindl.) Rchb.f. (1861) | *Polymeres* (Blume) J.J.Verm. & O'Byrne (2008) | D.L.Jones 3587 (CBG 8901443.1) | CNS_G05267* | 70 plastid genes \| ITS_ETS cistron |
| *Bulbophyllum maxillare* 2 (Lindl.) Rchb.f. (1861) | *Polymeres* (Blume) J.J.Verm. & O'Byrne (2008) | J.M.Taylor 284 (CANB) | CNS_G01299* | 70 plastid genes \| ITS_ETS cistron |
| *Bulbophyllum ocellatum* Cootes & M.A.Clem. (2011) | *Polymeres* (Blume) J.J.Verm. & O'Byrne (2008) | M.A.Clements 11603 (CANB 882133.1) | CNS_G05293* | 70 plastid genes \| ITS_ETS cistron |
| *Bulbophyllum rhodoglossum* Schltr. (1913) | *Polymeres* (Blume) J.J.Verm. & O'Byrne (2008) | T.M.Reeve 995 (CBG 8905750.1) | CNS_G07310* | 70 plastid genes \| ITS_ETS cistron |
| *Bulbophyllum wolfei* B.Gray & D.L.Jones (1991) | *Polymeres* (Blume) J.J.Verm. & O'Byrne (2008) | D.L.Jones4353 (CBG 8913157) | CNS_G01623* | ITS \| ycf1 |
| *Bulbophyllum radicans* F.M.Bailey (1897) | *Polymeres* Verm. & O'Byrne 2008 | Lockyer, R. 6 (CBG 8605880.1) | CNS_G05228* | 70 plastid genes \| ITS_ETS cistron |
| *Bulbophyllum schinzianum* Kraenzl. (1899) | *Ptiloglossum* Lindl. (1862) | M.A.Clements 11554 (CANB) | CNS_G01642* | 70 plastid genes \| ITS_ETS cistron |
| *Bulbophyllum intricatum* Seidenf. (1979) | *Racemosae* Benth. & Hook.f. (1883) | G.McCraith 83 (CANB) | CNS_G05202* | 70 plastid genes \| ITS_ETS cistron |
| *Bulbophyllum propinquum* Kraenzl. (1908) | *Racemosae* Benth. & Hook.f. (1883) | Orchid Research Group 7667 (CANB) | CNS_G07576* | 70 plastid genes \| ITS_ETS cistron |
| *Bulbophyllum saurocephalum* | *Saurocephalum* Schltr. (1912) | M.A.Clements 11496 (CANB 949470.1) | CNS_G05212* | 70 plastid genes \| ITS_ETS cistron |
| *Bulbophyllum baileyi* F.Muell. (1875) | *Sestochilos* (Breda) Benth. & Hook.f. (1883) | D.L.Jones18707c (CANB) | CNS_G00242* | ITS \| matK \| ycf1 |
| *Bulbophyllum gjellerupii* J.J.Sm. (1929) | *Sestochilos* (Breda) Benth. & Hook.f. (1883) | M.A.Clements 11594 (CANB 925027.1) | CNS_G05270* | 70 plastid genes \| ITS_ETS cistron |
| *Bulbophyllum lobbii* Lindl. (1847) | *Sestochilos* (Breda) Benth. & Hook.f. (1883) | Orchid Research Group 5166 (CANB) | CNS_G05229* | 70 plastid genes \| ITS_ETS cistron |
| *Bulbophyllum macranthum* Lindl. (1844) | *Sestochilos* (Breda) Benth. & Hook.f. (1883) | 952179 (CANB 952179.1) | CNS_G05452 | ITS_ETS cistron |
| *Bulbophyllum flavescens* (Blume) Lindl. (1830) | *Stachysanthes* (Blume) Averyanov (1994) | M.A.Clements 11548 (CANB) | CNS_G05280* | 70 plastid genes \| ITS_ETS cistron |
| *Bulbophyllum cambodianum* (H.Wendl. & Kraenzl.) Rolfe (1897) | *Trias* (Lindl.) J.J.Verm., Schuit. & de Vogel (2014) | R.Cowen 3077 (CANB) | CNS_G05275* | 70 plastid genes \| ITS_ETS cistron |
| *Bulbophyllum tripudians* C.S.P.Parish & Rchb.f. (1875) | *Tripudianthes* Seidenf. (1979) | Orchid Research Group 3081 (CANB) | CNS_G05249* | 70 plastid genes \| ITS_ETS cistron |
| *Bulbophyllum cylindrobulbum* Schltr. (1905) | *Uncifera* Schltr. (1912) | M.A.Clements 11200 (CANB 998220.1) | CNS_G05215* | 70 plastid genes \| ITS_ETS cistron |
| *Bulbophyllum* sp. |  | T.M.Reeve 1097 (CBG 8905768.1) | CNS_G07341 | ITS_ETS cistron |
| *Coelogyne flaccida* Lindl. |  | (WIS v0289172) |  | plastid genes \| ITS |
| *Dendrobium brunneum* Schuit. & P.B.Adams |  | Orchid Research Group 5485 (CANB) | CNS_G00781* | plastid genes \| ITS |
| *Dendrobium cunninghamii* Lindl. |  | B.P.J. Molloy 061/98 (CANB) | CNS_G02336* | plastid genes \| ITS |
| *Dendrobium fuscescens* (Griff.) Summerh. |  | Orchid Research Group 6920 (CANB) | CNS_G01021* | plastid genes \| ITS |
| *Dendrobium macropus* (Endl.) Rchb.f. |  | P.D.Ziesing 345 (CANB) | CNS_G02316* | plastid genes \| ITS |
| *Dendrobium moniliforme* (L.) Sw. |  | M.A.Clements 8262 (CANB) | CNS_G00882* | plastid genes \| ITS |
| *Dendrobium muricatum* Finet |  | M.A.Clements 3093 (CANB) | CNS_G02350* | plastid genes \| ITS |
| *Dienia ophrydis* (J.Koenig) Seidenf. |  | C.H.Broers 458 (CBG 9306410) | CNS_G01303* | plastid genes \| ITS |
| *Neottia cordata* (L.) Rich. |  | M.A.Clements 9865 (CANB) | CNS_G05314* | plastid genes \| ITS |
| *Nervilia concolor* (Blume) Schltr. |  | L.J.Roberts ORG3787 (CANB) | CNS_G03442* | plastid genes \| ITS |
| *Oberonia complanata* (A.Cunn.) M.A.Clem. & D.L.Jones |  | C.D.Kilgour 633 (CNS135322) | CNS_G00309* | plastid genes \| ITS |

# Supplementary material S2: Plastid genes included in analyses.

| accD | infA | psaB | psbH | rpl2 | rpoB | rps14 |
| --- | --- | --- | --- | --- | --- | --- |
| atpA | matK | psaC | psbI | rpl14 | rpoC1 | rps15 |
| atpB | orf42 | psaI | psbJ | rpl16 (in part) | rpoC2 | rps16 |
| atpE | petA | psaJ | psbK | rpl20 | rps2 | rps18 |
| atpF | petB (in part) | psbA | psbL | rpl22 | rps3 | rps19 |
| atpH | petD (in part) | psbB | psbM | rpl23 | rps4 | ycf1 |
| atpI | petG | psbC | psbN | rpl32 | rps7 | ycf2 |
| ccsA | petL | psbD | psbT | rpl33 | rps8 | ycf3 |
| cemA | petN | psbE | psbZ | rpl36 | rps11 | ycf4 |
| clpP | psaA | psbF | rbcL | rpoA | rps12 | ycf68 |

# Supplementary material S3: PCR reaction protocols.

PCR reactions were carried out in 20 µL volumes. ITS reactions consisted of 2.5 µL PCR buffer (200mM Tris HCl pH 8.4, 500mM KCl), 0.5 µL MgCl (25mM), 0.5 µL each of forward and reverse primer (10µM), 0.5 µL dNTPs (10mM), 1 µL DMSO, 0.9 µL BSA, 0.4 µL KAPA Taq DNA polymerase (5U/µL) (Kapa Biosystems, Wilmington, USA), 10.7 µL H_2_O and 2.5 µL gDNA (ca. 5 ng/µL). For the amplification of *mat*K and *ycf*1, PCR reactions were carried out with 4 µL 5x High-Fidelity Buffer, 0.8 µL each of forward and reverse primer (10µM), 0.4µL dNTPs (10 mM), 0.6µL DMSO, 0.25 µL iProof High-Fidelity DNA polymerase (5U/µL), (Thermo Fisher Scientific, Waltham, USA), 11.15 µL H_2_O, and 2 µL gDNA (ca. 5 ng/µL).

For ITS a touchdown PCR was carried out with an initial denaturation step at 94^°^C for 2 min, followed by 7 cycles of 94 ^°^C denaturation for 45 sec, 66 ^°^C annealing for 45 sec (reducing 1 ^o^C at each cycle) and 72 ^°^C extension for 1 min and 30 sec, with a final extension at 72 ^°^C for 5 min. For *mat*K, an initial denaturation step was carried out at 98 ^°^C for 45 sec followed by 35 cycles of 98 ^°^C denaturation for 10 sec, 60 ^°^C annealing for 45 sec and 72 ^°^C extension/elongation for 45 sec, with a final extension at 72 ^°^C for 10 min. For *ycf*1, PRC reactions were carried out with the same conditions as for *mat*K, except for the annealing temperature which was set to 63 ^°^C. PCR products were cleaned using exonuclease (ExoI) and alkaline phosphatase (FastAP) (Thermo Fisher Scientific, Waltham, USA) using 7.5µL of PCR product, 0.25µL of ExoI, 1 µL of FastAP and 1.25 µL of H_2_O incubated for 15 mins at 37 ^°^C, followed by 15 mins at 85 ^°^C.

# Supplementary material S4: Comparison of likelihood scores for divergence dating analyses with alternative clock and speciation and extinction models.

| log file | AICM likelihood | +/- | burnin | bootstrap replicates | delta_AICM |
| --- | --- | --- | --- | --- | --- |
| relaxed log normal \| Birth Death | 477294.182 | 3.6993 | 0 | 1000 |  |
| relaxed log normal \| Yule | 477309.447 | 3.9273 | 0 | 1000 | 15.2643 |
| strict \| Yule | 480530.16 | 2.6903 | 0 | 1000 | 3235.978 |
| strict \| Birth Death | 480531.156 | 2.4152 | 0 | 1000 | 3236.9736 |

# Supplementary material S5: Comparison of likelihood scores for three models of range evolution.

|  | LnL | numparams | d | e | AICc | AICc_wt |
| --- | --- | --- | --- | --- | --- | --- |
| BAYAREALIKE | -287.2 | 2 | 0.0048 | 0.053 | 578.5 | 0.88 |
| DEC | -289.2 | 2 | 0.0079 | 0.0064 | 582.6 | 0.12 |
| DIVALIKE | -301.7 | 2 | 0.009 | 0.0033 | 607.6 | 4.40E-07 |

#

# Supplementary material S6: Maximum likelihood phylogenetic reconstruction of *Bulbophyllum* based on the 70 gene complete plastid dataset with reduced sampling.


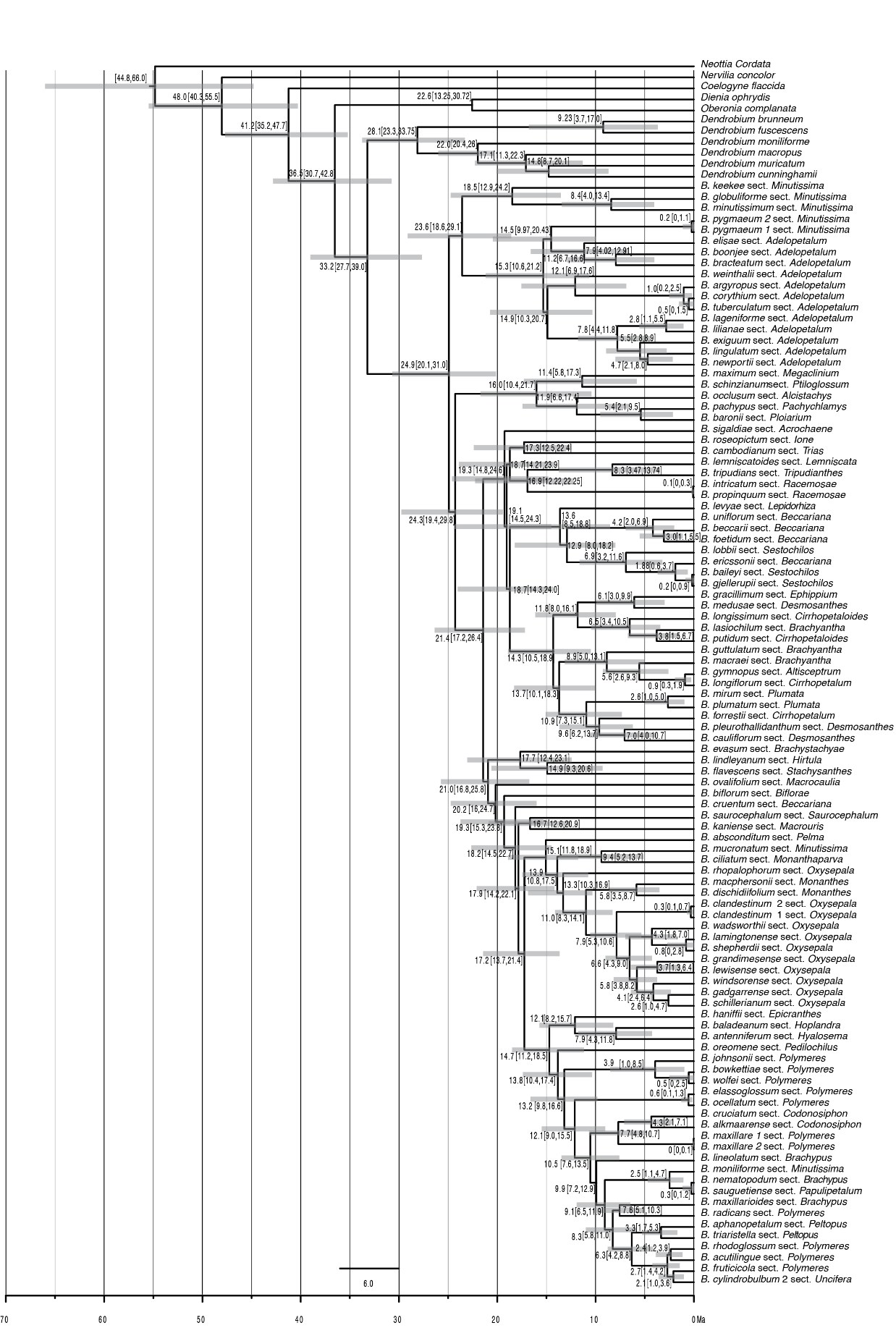


# Supplementary material S7: Maximum clade credibility chronogram for *Bulbophyllum* based on 70 plastid coding sequences, relaxed log normal clock and birth death prior. Divergence dates and 95% highest posterior density values are indicated adjacent to nodes. Grey bars indicate 95% highest posterior density.

# Supplementary material S8: Ancestral area reconstruction based on a the BAYAREALIKE model with pie charts at internal nodes representing marginal probabilities for alternative ancestral areas. Map shows geographic regions delineated in the analysis and legend shows color-coded geographic regions and shared ancestral ranges.

# Supplementary material S9: Range probabilities reconstructed for each node in the ancestral area reconstruction based on a the BAYAREALIKE model. Nodes IDs are presented in Supplementary Material S10.

| **Node ID** | **Range probabilities (%)** |
| --- | --- |
| node 106 | BC 31; BCD 16; BCE 9; BCG 9; ABC 9; BCDE 4; BCDG 4; ABCD 4; BCEG 2; ABCE 2; ABCG 2; BCDEG 1; ABCDE 1; ABCDG 1; CD 1; ABCEG 1 |
| node 107 | BC 70; BCD 17; BCE 3; BCG 3; ABC 3; BCDE 1; BCDG 1; ABCD 1 |
| node 108 | BC 73; BCD 20; BCE 2; BCG 1; ABC 1; C 1 |
| node 109 | BC 85; C 12; BCD 2 |
| node 110 | BC 83; C 10; BCD 4; CD 1 |
| node 111 | C 97; BC 2 |
| node 112 | BC 74; C 15; BCD 7; CD 2 |
| node 113 | BC 50; BCD 28; C 11; CD 9; BCE 1 |
| node 114 | C 98; CD 1; BC 1 |
| node 115 | C 90; CD 6; BC 3 |
| node 116 | CD 60; CDE 14; CDG 13; BCD 4; CDEG 3; C 1; BCDE 1; BCDG 1 |
| node 117 | CD 40; C 31; BC 13; BCD 10; CE 1; CDE 1; CDG 1; CG 1; BCE 1 |
| node 118 | BC 28; CD 25; BCD 24; C 20; BCE 1; CE 1 |
| node 119 | CD 80; C 19 |
| node 120 | CD 79; C 21 |
| node 121 | CD 89; CDE 10; D 1 |
| node 122 | CD 97; CDE 2 |
| node 123 | CD 89; C 8; CDE 1; BCD 1 |
| node 124 | CD 90; C 5; BCD 2; CDE 1 |
| node 125 | CD 89; C 5; BCD 3; CDE 1 |
| node 126 | CD 56; C 27; BC 7; BCD 6; CE 2; CDE 2 |
| node 127 | C 41; CD 39; BC 9; BCD 5; CE 2; CDE 1 |
| node 128 | C 90; CD 6; BC 2; CE 1 |
| node 129 | C 100 |
| node 130 | C 53; CD 35; BC 6; BCD 2; CE 2; CDE 1 |
| node 131 | CD 49; C 35; BC 7; BCD 5; CE 2; CDE 1 |
| node 132 | CD 55; C 28; BC 7; BCD 5; CE 2; CDE 2 |
| node 133 | CD 54; C 28; BC 6; BCD 5; CE 3; CDE 3 |
| node 134 | D 42; DE 37; CD 15; CDE 6 |
| node 135 | D 99; DG 1 |
| node 136 | D 95; DG 5 |
| node 137 | D 98; DG 2 |
| node 138 | D 84; DG 13; DE 1; CD 1 |
| node 139 | D 83; DE 8; CD 5; DG 3 |
| node 140 | D 41; DE 33; CD 18; CDE 7 |
| node 141 | DE 49; D 45; E 6 |
| node 142 | CD 49; CDE 25; DE 9; D 9; CE 6; C 2; E 1 |
| node 143 | CD 47; CDE 28; DE 14; D 11 |
| node 144 | CD 51; CDE 32; DE 9; D 8 |
| node 145 | CD 92; CDE 7 |
| node 146 | CDE 100 |
| node 147 | CD 58; CDE 41 |
| node 148 | CD 58; CDE 41; DE 1 |
| node 149 | C 100 |
| node 150 | CD 57; CDE 41 |
| node 151 | E 100 |
| node 152 | E 88; CE 5; DE 5 |
| node 153 | CD 54; CDE 44 |
| node 154 | CD 59; CDE 39 |
| node 155 | C 42; CG 22; CD 15; CDG 6; CE 5; CEG 2; BC 2; CDE 1; BCG 1; CDEG 1; BCD 1 |
| node 156 | CD 38; C 25; CDE 10; CE 8; CDG 4; BCD 3; CG 3; BC 2; CDEG 1; CEG 1; BCDE 1; BCE 1 |
| node 157 | CD 61; CDE 36; CDG 1; BCD 1 |
| node 158 | E 100 |
| node 159 | E 99 |
| node 160 | E 99 |
| node 161 | E 96; DE 2; CE 2 |
| node 162 | E 100 |
| node 163 | E 97; DE 2; CE 2 |
| node 164 | E 85; DE 7; CE 7; CDE 1 |
| node 165 | CDG 100 |
| node 166 | CDE 62; DE 13; CE 13; CD 5; CDEG 2; E 1; DEG 1; CEG 1 |
| node 167 | CDE 70; CD 13; DE 7; CE 6 |
| node 168 | CDE 76; CD 16; DE 4; CE 3; CDEG 1 |
| node 169 | CD 54; CDE 43; DE 1; CDG 1; D 1 |
| node 170 | C 56; CD 28; CE 9; CDE 5 |
| node 171 | CD 58; CDE 40; CDG 1 |
| node 172 | CD 60; CDE 38; CDG 1; CDEG 1 |
| node 173 | CD 62; CDE 37 |
| node 174 | CD 67; CDE 29; BCD 1 |
| node 175 | CD 62; CDE 36; BCD 1 |
| node 176 | CD 62; CDE 36; BCD 1 |
| node 177 | CD 49; CDE 28; CE 10; C 8; BCD 2; BCDE 1; BC 1; BCE 1 |
| node 178 | CD 42; CDE 25; CE 15; C 10; BCD 3; BC 2; BCE 1; BCDE 1 |
| node 179 | C 45; CE 24; CD 18; CDE 6; BC 2; BCE 1; BCD 1; AC 1 |
| node 180 | CE 32; CD 23; C 22; CDE 15; BC 2; BCE 2; BCD 1; BCDE 1 |
| node 181 | CD 37; CDE 25; CE 19; C 11; BC 2; BCD 2; BCE 2; BCDE 1 |
| node 182 | CD 35; CDE 24; CE 19; C 11; BC 3; BCD 2; BCE 2; BCDE 1; ACD 1 |
| node 183 | A 97; AC 1; AE 1; AD 1 |
| node 184 | A 81; AC 6; AE 5; AD 3; AG 1; AB 1; AF 1 |
| node 185 | A 80; AC 6; AE 6; AD 3; AG 1; AB 1; AF 1 |
| node 186 | A 57; AC 13; AE 11; AD 6; ACE 2; AG 2; AB 1; ACD 1; AF 1; ADE 1 |
| node 187 | CE 24; CDE 20; ACE 8; CD 7; ACDE 4; CEG 3; BCE 3; C 3; ACD 2; DE 2; AC 2; CDEG 2; CEF 2; BCDE 1; E 1; AE 1; CDEF 1; ADE 1; CDG 1; BC 1; ABCE 1; CG 1; ACEG 1; BCD 1; CDF 1 |
| node 188 | F 41; G 34; E 11; FG 9; EF 3; EG 2 |
| node 189 | E 36; EF 33; EG 26; EFG 3; F 2; G 1 |
| node 190 | E 72; EF 16; EG 7; CE 1; DE 1; EFG 1; AE 1 |
| node 191 | E 99 |
| node 192 | E 85; EG 15 |
| node 193 | E 90; EG 9 |
| node 194 | E 92; EG 6; EF 1 |
| node 195 | E 72; EF 16; EG 6; CE 2; DE 1; AE 1 |
| node 196 | E 95; EF 2; EG 1; CE 1 |
| node 197 | E 89; EF 6; EG 2; CE 1; DE 1 |
| node 198 | E 71; EF 18; EG 5; CE 2; DE 1; AE 1 |
| node 199 | E 71; EF 16; EG 6; CE 3; DE 1; AE 1; EFG 1 |
| node 200 | E 82; CE 5; EG 4; DE 3; AE 2; EF 1; BE 1 |
| node 201 | E 24; CE 18; EG 13; DE 7; CEG 6; CDE 4; AE 4; DEG 3; ACE 2; EF 2; BE 2; CDEG 1; AEG 1; CEF 1; BCE 1; ADE 1; ACEG 1; EFG 1; BEG 1 |
| node 202 | CE 26; CDE 13; E 11; DE 7; ACE 6; CEG 5; AE 3; EG 3; CEF 3; BCE 2; ACDE 2; CDEG 2; ADE 1; DEG 1; CDEF 1; BE 1; EF 1; ACEG 1; BCDE 1; DEF 1; AEG 1 |
| node 203 | CE 26; CDE 21; ACE 8; CEG 5; ACDE 4; BCE 3; CDEG 3; DE 3; CEF 2; CD 2; CDEF 2; BCDE 1; E 1; AE 1; ADE 1; ACD 1; ACEG 1; AC 1; ABCE 1; C 1; CDG 1; DEG 1; ACEF 1; EG 1 |
| node 204 | EG 8; FG 8; EF 7; EFG 7; G 4; CEG 4; F 4; CEF 4; CFG 3; CE 3; E 3; CG 3; CF 3; CEFG 3; BEG 2; BEF 2; BFG 2; BG 2; BE 2; BF 2; BEFG 2; BCE 1; BCG 1; BCEG 1; C 1; BCF 1; BCEF 1; BCFG 1; BCEFG 1; DEG 1; DEF 1; BC 1; DFG 1; AEG 1; AEF 1; DG 1; DEFG 1; DF 1; DE 1; AFG 1 |
| node 205 | EG 8; EF 7; EFG 6; CEG 6; CE 5; CEF 5; E 4; CEFG 4; BEG 3; FG 3; BEF 3; BE 3; BCE 2; CFG 2; CG 2; CF 2; BCEG 2; BEFG 2; BCEF 2; G 1; BCEFG 1; F 1; BFG 1; BG 1; BCG 1; BF 1; BCF 1; DEG 1; DEF 1; BCFG 1; BC 1; DE 1; CDE 1; AEG 1; C 1; AEF 1; CDEG 1; CDEF 1; AE 1; ACE 1; DEFG 1 |
| node 206 | BCE 8; CE 7; CEG 6; CEF 6; BCEG 6; BCEF 5; CEFG 3; BCEFG 3; BCG 3; BCF 2; BC 2; CG 2; CF 2; CFG 2; BE 2; BEG 2; BCFG 2; BCDE 2; BEF 2; CDE 2; CDEG 1; BCDEG 1; ABCE 1; CDEF 1; EG 1; ACE 1; BCDEF 1; EF 1; C 1; BEFG 1; ACEG 1; EFG 1; ACEF 1; ABCEG 1; ABCEF 1; CDEFG 1; BCD 1; BCDG 1; BG 1; CDG 1; BCDF 1; E 1 |
| node 207 | BC 45; C 31; BCE 4; CE 3; BCG 2; BCF 2; BCD 2; ABC 2; CG 2; CF 2; CD 1; AC 1 |
| node 208 | BCE 9; CE 7; BCEG 6; CEG 5; BCEF 5; CEF 4; BCDE 4; CDE 3; BCEFG 3; CEFG 3; ABCE 2; BCDEG 2; BC 2; CDEG 2; BCDEF 2; BCG 2; ACE 2; CDEF 2; BCF 2; ABCEG 1; ACEG 1; CG 1; ABCEF 1; ACEF 1; CF 1; BCD 1; CDEFG 1; BCFG 1; ABCDE 1; CFG 1; BCDG 1; C 1; ACDE 1; BCDF 1; ABC 1; CDG 1; ACEFG 1; CD 1; CDF 1; ABCG 1; ACDEG 1; BE 1; BEG 1; ACDEF 1; ABCF 1 |
| node 209 | BCE 6; BCDE 5; CDE 5; CE 5; BCEG 4; CEG 4; BCDEG 4; CDEG 4; BCEF 4; CEF 4; BCDEF 3; CDEF 3; ABCE 3; ACE 3; BCEFG 3; ABCDE 3; CEFG 3; ACDE 2; ABCEG 2; ACEG 2; CDEFG 2; ABCEF 2; ACEF 2; ACDEG 2; ACDEF 2; ACEFG 1; BCD 1; BCDG 1; BCDF 1; BCG 1; CDG 1 |

# Supplementary material S10: Nodes IDs for ancestral area reconstruction range probabilities.
